# A modified Mediterranean-style diet enhances brain function via specific gut-microbiome-brain mechanisms

**DOI:** 10.1080/19490976.2024.2323752

**Published:** 2024-03-06

**Authors:** Gwoncheol Park, Saurabh Kadyan, Nathaniel Hochuli, Julie Pollak, Bo Wang, Gloria Salazar, Paramita Chakrabarty, Philip Efron, Julia Sheffler, Ravinder Nagpal

**Affiliations:** a The Gut Biome Lab, Department of Health, Nutrition, and Food Sciences, College of Education, Health, and Human Science, Florida State University, Tallahassee, FL, USA; b Department of Health, Nutrition, and Food Sciences, College of Education, Health, and Human Science, Florida State University, Tallahassee, FL, USA; c Department of Chemistry and Chemical Engineering, Florida Institute of Technology, Melbourne, FL, USA; d Center for Translational Research in Neurodegenerative Diseases, Department of Neuroscience, University of Florida, Gainesville, FL, USA; e Sepsis and Critical Illness Research Center, Department of Surgery, University of Florida College of Medicine, Gainesville, FL, USA; f Center for Translational Behavioral Science, Department of Behavioral Sciences and Social Medicine, Florida State University College of Medicine, Tallahassee, FL, USA

**Keywords:** Alzheimer’s disease, gut-brain axis, ketogenic diet, Mediterranean diet, metabolome, microbiome, neurocognition, neurodegenerative disease, neuroinflammation

## Abstract

Alzheimer’s disease (AD) is a debilitating brain disorder with rapidly mounting prevalence worldwide, yet no proven AD cure has been discovered. Using a multi-omics approach in a transgenic AD mouse model, the current study demonstrated the efficacy of a modified Mediterranean-ketogenic diet (MkD) on AD-related neurocognitive pathophysiology and underlying mechanisms related to the gut-microbiome-brain axis. The findings revealed that MkD induces profound shifts in the gut microbiome community and microbial metabolites. Most notably, MkD promoted growth of the *Lactobacillus* population, resulting in increased bacteria-derived lactate production. We discovered elevated levels of microbiome- and diet-derived metabolites in the serum as well, signaling their influence on the brain. Importantly, these changes in serum metabolites upregulated specific receptors that have neuroprotective effects and induced alternations in neuroinflammatory-associated pathway profiles in hippocampus. Additionally, these metabolites displayed strong favorable co-regulation relationship with gut-brain integrity and inflammatory markers, as well as neurobehavioral outcomes. The findings underscore the ameliorative effects of MkD on AD-related neurological function and the underlying gut-brain communication via modulation of the gut microbiome-metabolome arrays.

## GRAPHICAL ABSTRACT

**Figure:**
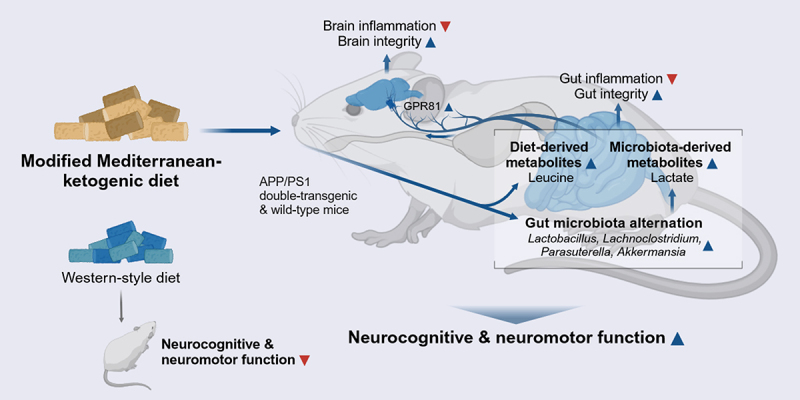

## Introduction

Alzheimer’s disease (AD) is the predominant form of dementia, comprising 60–80% of reported cases. AD ranks as the seventh leading cause of death worldwide, and the risk of developing AD doubles every five years for those over the age of 65. The multifactorial complexity of AD etiology, with genetic and environmental risk factors, makes it improbable that any single drug or intervention will effectively treat or cure AD for all patients. Nevertheless, emerging evidence shows that lifestyle behaviors, such as consuming a nutritious diet, may promote healthy aging and reduce the risk of AD.^1^ Notably, one-third of AD cases are linked to modifiable risk factors that can be alleviated through dietary and lifestyle adjustments.^2^ Thus, dietary and lifestyle interventions are a promising pathway for reducing AD incidence worldwide.

Diet provides nutrients and energy to the host and shapes the gut environment through direct interactions with the microbiota. The gut microbiome, characterized by its dynamic complexity and myriad symbiotic relationships among microbes or between microbes and the host, demonstrates remarkable flexibility and can be readily modulated. It exhibits daily cyclical fluctuations in composition, and dietary modification can induce significant microbiome changes within a single day.^3^ These alterations in the intestinal microbiota subsequently impact the gut microecological environment, leading to shifts in microbiota-derived metabolites. However, diet effects are not limited to the gut. The subsequent changes triggered by dietary shifts in the gut can profoundly affect the brain through a network of intricate connections between the two organs, including pathways involving the vagus nerve, immune system responses, and bacterial metabolites and byproducts. This bidirectional communication network, often referred to as the ‘gut-microbiome-brain’ axis, assumes a pivotal role in maintaining homeostasis in the gastrointestinal tract and central nervous system.^4^ Studies have demonstrated the crucial role of microbial communities and their function in neurodegenerative disease,^5^ as well as the ameliorative effects of modulating the gut microbiome on neurodegeneration.^6^ Specifically, dysbiosis in the gut leads to an increase in lipopolysaccharides (LPS) in systemic circulation, which stimulate the inflammatory signaling pathway, contributing to neurodegeneration and neuronal death.^7^ Intriguingly, recent studies have revealed that amyloid-beta peptide (Aβ), known for its critical role in AD pathology, possesses antimicrobial activity. Aβ increases during brain infection by pathogen from the gut, acting as a double-edged sword.^8^ Amyloids derived from bacterial organisms have also been suggested as provoking factors for the deposition of Aβ in AD by stimulating the immune system and inducing systemic inflammation.^9^

The Western-style dietary pattern (WD), also referred to as an average American diet, is characterized by its low fiber content and high levels of refined sugar and saturated fat. As the name implies, it reflects the typical dietary pattern of industrialized countries and is associated with obesity, metabolic disease, and cardiovascular disease. The WD may be a contributing factor in triggering AD-related pathology by inducing systemic inflammation and impairing the gut-blood-brain barrier (BBB), thereby leading to neuroinflammation.^10^ On the other hand, the Mediterranean diet (MD), rich in fiber and unsaturated fats, has been scientifically endorsed as a beneficial approach in preventing and managing various diseases, promoting healthy aging,^11^ and effectively modulating the gut microbiome and metabolite profiles to reduce the risk of cognitive decline or AD.^12^ Additionally, the Ketogenic diet (KD), characterized by high fat and very low carbohydrate intake, induces a state of ketosis. This state offers ketones as an alternative metabolic precursor to glucose in the brain, particularly in milieus where brain cells face challenges in using glucose as an energy source due to systemic metabolic impairments (e.g., brain insulin resistance). Ketones have been shown to confer a neuroprotective role and improvements in cognitive function following ketogenic interventions by enhancing brain energy metabolism.^13^ While it shows promise as a dietary intervention to improve cognitive symptoms at the prodromal stage of AD,^14^ adherence to the KD can be challenging due to its extreme nature, particularly for older adults who have elevated risk for micronutrient deficiencies, impaired bone health, and liver diseases.^15–17^ To this end, we have recently demonstrated the benefits of combining both MD and KD to mitigate the adverse impacts of the KD while maximizing advantages from both diets and have demonstrated benefits for cognitive functioning.^18–20^ Propelled by our preceding studies,^18–20^ we herein comprehensively examine the effects of a modified Mediterranean-ketogenic diet (MkD) versus WD on entero-metabolic and neurocognitive health in a preclinical AD model. To elucidate the underlying mechanisms, we executed a high-throughput multi-omics approach, including metagenomics and metabolomics, to assess the causal mechanisms of dietary-modulated microbiome and metabolite signatures in gut-brain integrity, inflammatory arrays, and AD-related neurocognitive features. Finally, we identified specific microbes and microbial metabolites that demonstrate ameliorative effect on neuroinflammatory and neurodegenerative arrays in the context of AD neuropathology.

## Results

### MkD intervention prevents metabolic dysregulation in an AD model

**Figure 1.**
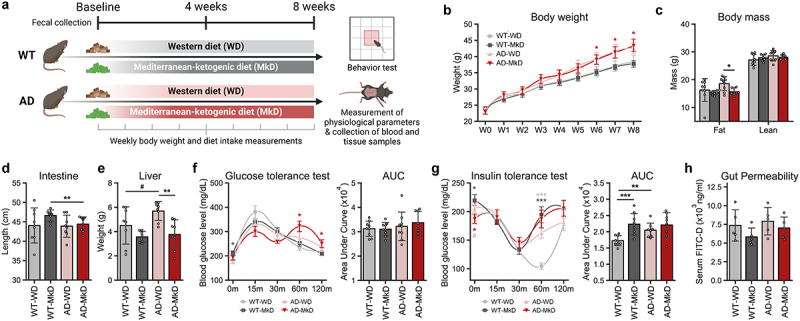
A Mediterranean-ketogenic diet, compared to a standard Western-style diet, improves body-composition in APP/PS1 mice. (a) Schematic study design. (b) Weekly changes in bodyweight. (c) Differences in body mass (%) were evaluated in terms of total fat and lean mass per group. Comparison of (d) total gastrointestinal length and (e) liver weight. Blood glucose level changes in response to (f) oral glucose tolerance test and (g) intraperitoneal insulin tolerance test. The area under curve (AUC) was calculated as an index of glucose tolerance or insulin tolerance for each test. (h) Gut permeability measured by FITC-dextran (*n*=5/group). See also Figure S1. Data are presented as mean ± SD. Significance was determined by the one-way ANOVA test with post-hoc Dunn’s test. *n*=8–9 per group (except for gut permeability test). #p<0.1; *p<0.05; **p<0.01; ***p<0.001. For the line plots, the significance between groups was differentiated by color. Light gray color represents the difference between WT-WD and AD-WD, dark gray; WT-WD and WT-MkD, and red; WT-MkD and AD-MkD. WT: wild-type; AD: Alzheimer’s disease (APP/PS1 transgenic) mice; MkD: Mediterranean-ketogenic diet; WD: Western-style diet.

Through a 12-week dietary intervention with MkD vs. WD in transgenic APP/PS1 (AD) versus wild-type (WT) mice, we assessed the physiological parameters to determine the effects of the diets on body-composition and metabolic health. Overall, the AD mice exhibited higher bodyweight compared to the WT counterparts, primarily due to increased food intake starting from week 3, which continued until the study endpoint (Figure 1b, Figure S1A,B). We did not observe any significant differences in weight gain between the two diets; however, a significant difference in body-composition was observed. Specifically, in AD mice, MkD resulted in a significantly lower fat mass compared to the WD-fed group; and a similar pattern was observed in WT mice (Figure 1c). Furthermore, we noticed a tendency for longer intestinal length in the MkD-fed mice, while liver weight was higher in the WD-fed mice, particularly in the AD group (Figure 1d,e). Cecum weight remained relatively consistent across all groups (Figure S1E). During the glucose tolerance test (GTT), MkD-fed WT mice exhibited significantly elevated fasting glucose levels. Although the area under the curve (AUC) showed no significant difference between the groups, the peak post-prandial blood glucose level was higher in WD-fed mice compared to MkD-fed mice in both genotypes (Figure 1f, Figure S1C). Notably, we observed a higher AUC in the insulin tolerance test (ITT) in MkD-fed mice, with the difference being significant, especially in WT mice. An interesting observation in the ITT assessments was that the serum glucose level in WT-WD mice reached its lowest point 60 minutes after insulin injection, while all other groups reached this point at 30 minutes. Additionally, the WD-fed mice in both groups took relatively longer time to reach their original fasting glucose level (Figure 1g, Figure S1D). Furthermore, compared to WD, the MkD intervention consistently showed lower gut epithelial permeability in both genotypes (Figure 1h).

### MkD induces improvements in neurobehavioral function

**Figure 2.**
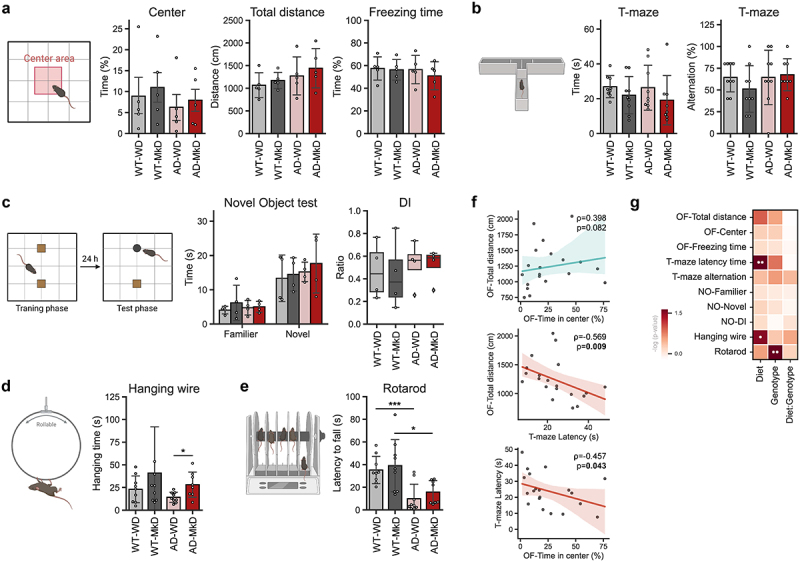
Mediterranean-ketogenic diet, versus a standard Western-style diet, improves neurocognitive and behavioral function in APP/PS1 mice. (a) Results of the open-field test, including time spent in the center area, total traversed distance, and freezing time (*n*=5/group). (b) Results of the T-maze spontaneous test, including the average time spent making decisions and the alternation score, which counts the alternative decisions compared to the previous trial (*n*=8–9/group). (c) Time spent near familiar and novel objects, along with the discrimination index value in the novel object test (*n*=4/group). (d) Time spent on the hanging wire (*n*=8–9/group). (e) Latency to fall on the rotating rod (*n*=8–9/group). (f) Correlation between behavioral outcomes using Spearman’s correlation. (g) Heatmap illustrating the differences in behavior assays outcomes on genotype and diet, as determined using a two-way ANOVA model (● *p*<0.1; ●● *p*<0.05). See also Figure S1. Data are presented as mean ± SD. Significance was determined by the one-way ANOVA test with post-hoc Dunn’s test. *p<0.05; ***p<0.001. WT: wild-type; AD: Alzheimer’s disease (APP/PS1 transgenic) mice; MkD: Mediterranean-ketogenic diet; WD: Western-style diet.

To explore the impact of diet on neurobehavioral and neuromuscular activities, we subjected the mice to a battery of behavioral assays. Overall, MkD showed a beneficial influence on various behavioral domains in mice, including exploratory behavior, anxiety levels, memory, and motor performance (Figure 2). Compared to WD counterparts, MkD-fed mice exhibited higher time spent in the center area and traversed greater distances in the open-field test (OPT), indicating modestly improved exploratory activity and lower anxiety-like behavior (Figure 2a). Spatial working memory, assessed by alternation score in the T-maze test, did not show a statistically significant difference between the two groups; however, the MkD-fed mice exhibited significantly reduced latency time, suggesting improvement in neurocognitive processing or task-specific adaptation, leading to better/faster decision-making (Figure 2b,f). Analogously, the discrimination index (DI), which reflects the object recognition and discrimination ability, in MkD-fed mice showed a tendency to explore more for both familiar and novel objects (Figure 2c). As widely recognized and expected in this model, AD mice displayed compromised neuromuscular ability, evidenced by shorter hanging time on the wire and decreased endurance on the accelerating rotating rod (Figure 2d–f). However, MkD-fed mice, particularly the AD mice, demonstrated significant improvement in the hanging-wire test and showed positive trends in the rotarod test (Figure 2d–e). In the location-memory test, which assesses spatial memory and discrimination ability, the discrimination ability of mice remained unchanged by the diet, though WT-MkD mice exhibited relatively better ability compared to WT-WD mice (Figure S1F). Similarly, all groups showed similar grip-strength, which measures muscular strength and motor function (Figure S1G). To validate consistency between the neurocognitive and behavioral tests, we calculated correlations among the results and found intriguing and strong associations (Figure 2f). For instance, mice that spent more time in the center area explored greater distance in the OPT (ρ = 0.398, p-value = 0.082) and spent less time making decisions on the T-maze (ρ=-0.457, p-value = 0.043). Additionally, mice that spent less time making decisions on the T-maze exhibited more active travel in the OPT (ρ = 0.569, p-value = 0.009). Taken together, these data indicate that MkD may benefit behavioral and neuromotor abilities compared to WD, particularly in AD mice.

### MkD induces distinct and positive gut microbiome modulation compared to WD intervention

**Figure 3.**
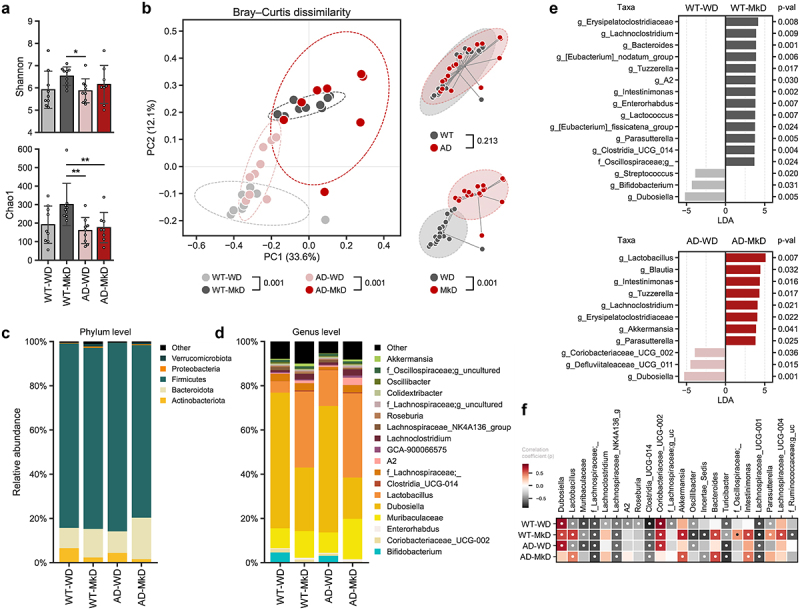
Mediterranean-ketogenic diet, versus standard Western-style diet, induces distinct and beneficial arrays of gut microbiome diversity and composition in APP/PS1 mice. (a) Alpha-diversity was determined using the Shannon index and Chao1 index. Significance between groups was calculated using the non-parametric Kruskal-Wallis test. (b) PCoA analysis based on Bray-Curtis dissimilarity was used to represent the beta-diversity of each group, genotype, and diet. Significance was calculated using PERMANOVA with 999 random permutations. Relative abundance of bacterial microbiome composition at the (c) phylum and (d) genus level. (e) Bacterial microbiome composition differences between groups were analyzed using the linear discrimination analysis (LDA) effect size (LEfSe) algorithm (LDA>3.0, p-value<0.05). (f) The heatmap represents the taxa changes over time for each group. Spearman’s correlation was used, and significantly correlated taxa (*p*<0.05) are indicated with a ‘●’ sign. See also Figure S3. *n*=8–9 per group. Data are presented as mean ± SD. *p<0.05; **p<0.01. WT: wild-type; AD: Alzheimer’s disease (APP/PS1 transgenic) mice; MkD: Mediterranean-ketogenic diet; WD: Western-style diet.

Propelled by the observed favorable outcomes of MkD on neurobehavior and metabolic features, we examined the diet-specific changes in the gut bacterial and fungal microbiomes, aiming to identify potential underlying mechanisms (Figure 3, Figure S2–5). In terms of the bacterial microbiome community, we observed slightly higher microbial alpha-diversity in MkD-fed mice from both genotypes, with a markedly distinct gut microbiome structure compared to WD-fed counterparts, resulting in distinct diet-specific beta-diversity clusters in the PCoA analysis (Figure 3a,b). Indeed, the mice showed substantially different microbiome composition profiles based on their diet. At the phylum level, MkD-fed mice had an increased abundance of Bacteroidota and reduced Actinobacteriota (Figure 3c). The downstream genus level comparison and the Linear Discriminatory Analysis (LDA) effect size (LEfSe) analysis revealed higher abundance of bacterial genera *Lactobacillus*, *Akkermansia*, g_*Erysipelatoclostridiaceae*, *Lachnoclostridium*, *Intestimonas*, and *Parasuterella*, while *Bifidobacterium* and *Dubosiella* were lower, in MkD-fed mice compared to WD-fed counterparts (Figure 3d,e). Specifically, *Lactobacillus*, *Akkermansia*, *Bacteroides*, and *Intestimonas* gradually increased over time in MkD-fed mice, whereas *Dubosiella* elevated more significantly, and *Lactobacillus* decreased markedly over time in WD-fed mice (Figure 3f, Figure S3D). The PICRUSt-inferred prediction of the microbiome’s functional readout also showed distinct arrays in MkD-fed vs. WD-fed WT mice, but no significant differences were seen in the AD group. However, several functional pathways showed identical patterns by diet in both genotypes. The abundance of KEGG pathways associated with D-alanine metabolism, glycosaminoglycan degradation, steroid hormone biosynthesis, and pathogen infection were more abundant in MkD mice, whereas the pathways related to the metabolism of amino acids (AAs; i.e., histidine, phenylalanine, tyrosine, and tryptophan) were more prevalent in WD group. Notably, MkD-fed mice showed less gene families annotated to the insulin signaling pathway compared to WD-fed mice (Figure S5).

Since changes in diet or bacterial microbiome may influence resident fungal flora, we also assessed the mycobiome (fungal microbiome) community. As such, we did not observe statistically significant differences or patterns in terms of the mycobiome diversity and composition based on the two diets (Figure S2A–B). Across all groups, the gut mycobiome was dominated by the Ascomycota phylum, and at the genus level, *Wickerhamomyces* and Saccharomycetales genera prevailed. Only the *Ophiostoma* genus showed significantly higher abundance in WT-WD-fed mice compared to WT-MkD-fed mice, but not in AD mice (Figure S2C–E). Over time, all major genera were constantly increased or decreased in all groups, although an unknown genus within the Ascomycota phylum was more rapidly decreased in MkD-fed mice whereas an unknown genus in Eurotiales order was more rapidly increased in WD-fed mice (Figure S2F, Figure S4D). Furthermore, while the two-way ANOVA test revealed that *Aspergillus*, *f_Mrakiaceae*, *Candida*, and *Saccharomyces* differed significantly by diet, these genera were almost depleted in all groups, regardless of their diets (Figure 5a, Figure S2F).

### MkD induces distinct fecal and serum metabolomics signatures by fostering specific neurotransmitter arrays

**Figure 4.**
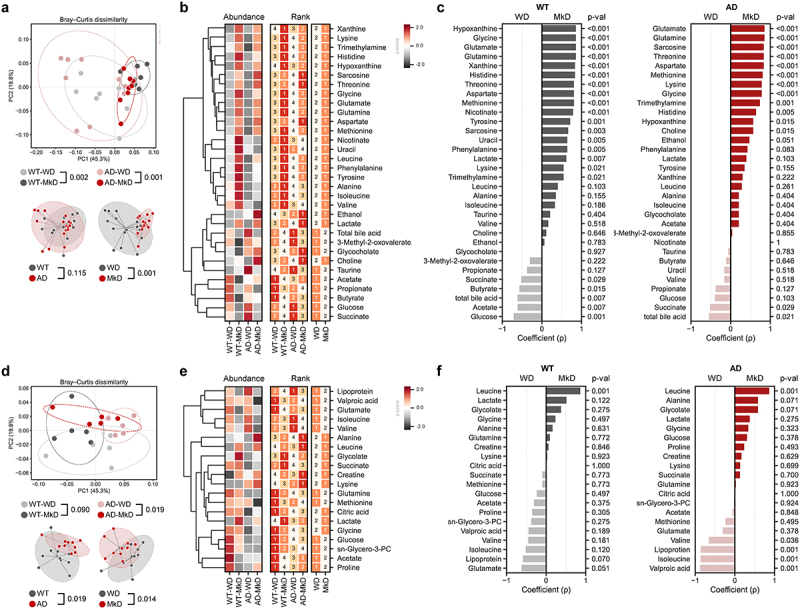
Mediterranean-ketogenic diet, versus standard Western-style diet, distinctly modulates the gut and blood metabolome in APP/PS1 mice. PCoA analysis of (a) fecal and (d) serum metabolites (untargeted global metabolome) was conducted based on Bray-Curtis dissimilarity, representing the beta-diversity of each group, genotype, and diet. Significance was calculated using PERMANOVA with 999 random permutations. The abundance of metabolites and the ranking of groups or diets by their average abundance are shown for (b) fecal and (e) serum metabolites. For the abundance heatmap, z-scores were calculated based on the average abundance of each group. Dendrograms were generated using hierarchical clustering results with the average linkage method, based on Bray-Curtis dissimilarity. Spearman’s correlation results between diets within each genotype are shown for (c) fecal and (f) serum metabolites. See also Figure S6. *n*=8–9 per group for fecal metabolome and *n*=5 per group for serum metabolome. WT: wild-type; AD: Alzheimer’s disease (APP/PS1 transgenic) mice; MkD: Mediterranean-ketogenic diet; WD: Western-style diet.

**Figure 5.**
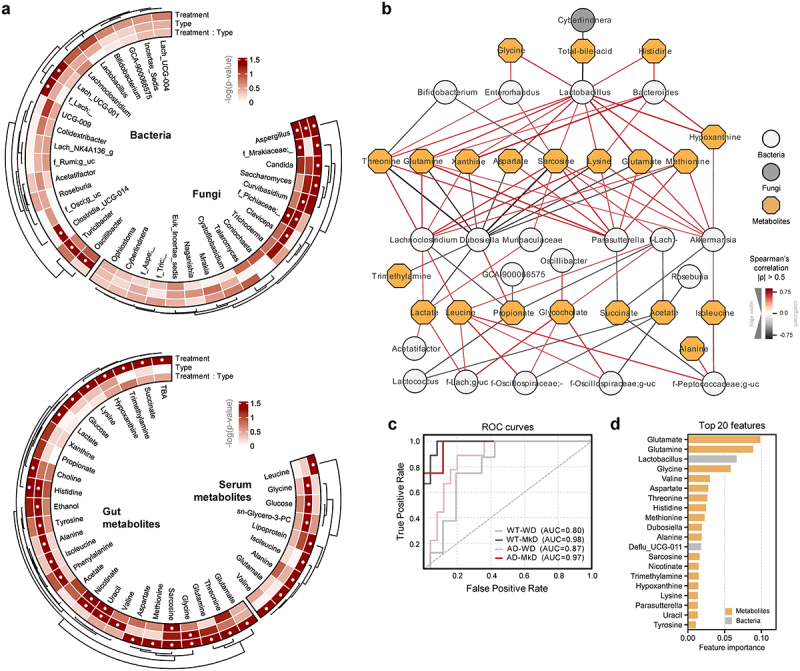
Mediterranean-ketogenic diet, versus standard Western-style diet, distinctly shapes the gut microecological niche via modulating the multi-omics arrays of host microbe-metabolite co-regulation interactions. Circular heatmap illustrating the differences in dominant (a) bacterial and fungal genera and gut and serum metabolites based on genotype and diet, as determined using a two-way ANOVA model. P-values were -log transformed and used for visualization. The dendrogram was generated using hierarchical clustering results with the average linkage method, based on Bray-Curtis dissimilarity. Significant taxa (*p*<0.05) are indicated with a ‘●’ sign. (b) Microbiota-metabolites co-occurrence network. Circular nodes represent genera (white: bacteria, gray: fungi), while yellow octagon nodes represent metabolites. Only significant links are shown here (Spearman’s rank correlation coefficient (ρ) >0.5; Benjamini–Hochberg corrected *p*<0.05). Red links denote positive correlation and black links indicate negative correlation, with line thickness corresponding to the correlation coefficient value. A random-forest prediction model between groups based on combined abundance data of microbiome and metabolome. (c) Receiver operating characteristic (ROC) curve depicts the classification accuracy, while (d) a bar graph highlights the top 20 most strongly predictive genera and metabolites based on relative importance scores. WT: wild-type; AD: Alzheimer’s disease (APP/PS1 transgenic) mice; MkD: Mediterranean-ketogenic diet; WD: Western-style diet.

Metabolites, which are produced during digestion and by the gut microbiota, represent an important factor in evaluating the effects and underlying mechanisms of the MkD. Certain dietary metabolites not only regulate gut health, but also exert direct effects on the immune system and brain. Given the substantial differences observed in the gut microbiome following the two dietary interventions, we performed global untargeted metabolomics to measure microbiota-derived metabolites in both feces and serum. Overall profiles of metabolites clearly varied according to the diet (Figure 4). Specifically, fecal metabolite profiles demonstrated stronger predictive power compared to bacterial and fungal community profiles when employing the Random Forest model with merged abundance data of microbiome and metabolomics (Figure 5c,d). Each group was successfully classified with high accuracy (AUC = 0.80–0.98). Notably, among the 20 most prominent features, 18 were metabolites, providing strong evidence that the diet effect was more pronounced in metabolites (Figure 5c,d). Certainly, the PCoA analysis based on the relative abundance of metabolites from the feces and serum showed distinct clustering signatures by diet, and notable differences were observed between WT and AD mice in serum metabolomics (Figure 4a,d). In the feces, most of the metabolites were more abundant in MkD-fed mice, with AAs, lactate, and purines being particularly prominent. It is noteworthy that precursors for neurotransmitters and neuroactive metabolites, including glycine, tyrosine, and histidine, were all significantly lower in AD mice but were markedly increased with MkD. Furthermore, excitatory neurotransmitter glutamate was higher in the MkD group, while glycine and sarcosine also exhibited an increase with MkD. Meanwhile, total bile acids (TBAs), several SCFAs, glucose, and succinate showed elevated levels with the WD (Figures 4b,c, 5A, Figure S6A). However, in the serum, relatively fewer metabolites were found to be more abundant following MkD. Leucine, lactate, alanine, and glycine were increased with MkD as in the feces, whereas glutamate and isoleucine displayed an inverse prevalence, being more prevalent with the WD diet. Additionally, lipoprotein and valine levels were higher in WD-fed mice (Figure 4e,f, Figure S6B).

We also applied co-occurrence network analysis to evaluate the associations between gut microbes and fecal-serum metabolites, aiming to gain insight as to how metabolites are modified by altered microbiota (Figure 5b). The majority of AAs, including threonine, glutamine, glutamate, and methionine, along with its metabolite sarcosine, exhibited positive correlations with *Lactobacillus*, *Lachnoclostridium*, *Parasutterella*, and *Akkermansia*, all of which were enriched following MkD. Particularly, the *Lactobacillus* genus coexisted with most metabolites increased by MkD and had a mutually exclusive relationship with TBAs. Conversely, *Dubosiella*, which was more prevalent in WD-fed mice, predominantly exhibited negative correlations with metabolites enriched by MkD. Notably, lactate demonstrated a positive correlation with microbiota belonging to the Lachnospiraceae family, which are estimated to utilize lactate, such as *Lachnoclostridium*, *Lactococcus*, and *g_uncultured*. Leucine also showed a mutual ascent co-regulation network with several microbial taxa belonging to families Lachnospiraceae and Oscillospiraceae (Figure 5b).

### MkD improves gut and brain inflammatory profiles, which strongly correlate with neurocognitive improvements

**Figure 6.**
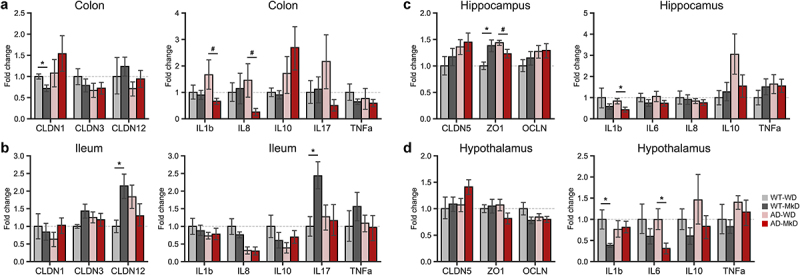
Mediterranean-ketogenic diet, versus standard Western-style diet, ameliorates gut and brain barrier integrity and lowers intestinal and neuronal inflammation in association with improved neurocognitive and behavioral functions in APP/PS1 mice. Gene expression of tight-junction proteins (CLDN1, CLDN3, and CLDN12) and inflammatory markers (IL-1β, IL-8, IL-10, IL-17, and TNF-α) in (a) colon and (b) ileum. Gene expression of tight-junction proteins (CLDN5, ZO-1, and OCLN) and inflammatory markers (IL-1β, IL-6, IL-8, IL-10, and TNF-α) in (c) hippocampus and (d) hypothalamus. Data are presented as mean ± SD. Significance was determined by unpaired t-tests between WT-WD and WT-MkD, as well as between AD-WD and AD-MkD. See also Figure S7-S8. *n*=6–8 per group. #p<0.1; *p<0.05. WT: wild-type; AD: Alzheimer’s disease (APP/PS1 transgenic) mice; MkD: Mediterranean-ketogenic diet; WD: Western-style diet.

Enhanced neurocognitive and neuromuscular activity, along with substantial modulation of the gut microbiome and fecal-serum metabolites, prompted us to investigate the state of epithelial barrier integrity and inflammation in both the gut and brain niches. To this end, we measured mRNA expression of genes encoding tight-junction proteins and inflammatory markers and determined their correlation with neurocognitive outcomes to understand potential underlying relationships. The distal part of small intestine (ileum) and large intestine (colon) were used and hippocampus and hypothalamus (regions damaged in the early stage of AD, leading to memory impairment and behavioral alternation) were chosen for gene quantification.^21,22^ We found that the MkD intervention tended to increase the expression of transmembrane protein Claudin (CLDN) 12, which regulates Ca^2+^ absorption and homeostasis, in both the ileum and colon, although a reverse trend occurred in AD mice. Furthermore, CLDN12 expression was positively correlated with motor functions (Figure 6a,b, Figure S7D–E). Interestingly, CLDN3, which directly influences junction tightness, also showed a positive association with motor functions, although its expression was not significantly different among groups. In the hippocampus and hypothalamus of MkD-fed mice, we found increased expression of CLDN5, the most enriched tight-junction protein implicated in neurodegenerative disorders. While zonulin (ZO)-1 was significantly increased in the hippocampus of WT-MkD mice compared to their WD counterparts, the AD groups exhibited reverse trends (Figure 6c,d, Figure S7A). Concomitant to improvement in barrier integrity, MkD also predominantly reduced the expression of pro-inflammatory mediators in both the gut and brain. We found a strong decline in interleukin (IL)-1β in the colon and hippocampus after MkD intervention, and this decrease negatively correlated with locomotor activity (Figure 6a,c Figure S7A–B). Additionally, MkD led to the downregulation of IL-6, known for its implication in neurodegeneration of the hippocampus and hypothalamus. Notably, lower hippocampal IL-6 expression was associated with higher locomotor activity (Figure 6c,d, Figure S7B). A constant marginal decrease in IL-8 was observed in the ileum and hippocampus after MkD, with its level inversely correlated with locomotor activity and proportionally related to latency time in the T-maze test (Figure 6b,c, Figure S7B–C). Tumor necrosis factor (TNF)-α also tended to be less expressed following MkD in the colon and hypothalamus, and the reduction in colonic TNF-α correlated with favorable outcomes in the OPT (Figure 6a,d, Figure S7A–B). Though there was a reverse trend between WT and AD mice, we found decreased IL-17 in the gut following MkD. On the other hand, IL-10 was increased in the gut but decreased in the brain after MkD in AD mice (Figure 6a–d). Based on these findings, in order to validate the transcriptional responses of key tight-junction proteins (CLDN5 and ZO-1) and inflammatory markers (IL-1β, IL-6, and TNF-α) to MkD and to assess if they translate into similar protein levels as their mRNA transcripts in the brain, the protein levels of these markers were quantified. Overall, cytokine levels exhibited similar expression patterns, decreasing in MkD-fed mice compared to WD-fed mice. Particularly, TNF-α showed a significant decrease after the MkD intervention in AD mice. However, tight-junction proteins demonstrated a reverse trend compared to their transcriptional responses, although ZO-1 levels were higher in AD-MkD mice compared to AD-WD mice (Figure S10A–B).

### Lactate and leucine, key metabolites, potentially mediate ameliorative effects of MkD on AD pathology

**Figure 7.**
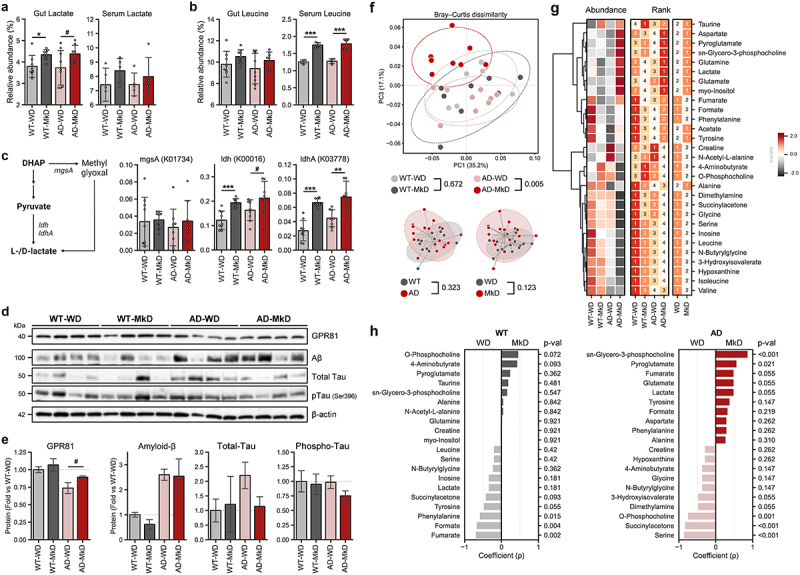
A Mediterranean-ketogenic diet increases lactate and leucine levels in both the gut and blood, which upregulate lactate receptor and induce changes in brain metabolome in APP/PS1 mice. (a) Relative abundance of gut and serum lactate. (b) Relative abundance of gut and serum leucine. (c) Bacterial biosynthesis pathway and associated genes for lactate and the predicted relative abundance of genes (KO orthology). (d) Protein levels of lactate receptor (GPR81) and AD biomarkers (Aβ and total-/phosphorylated-tau) in brain were quantified by Western blot (*n*=4/group). (e) The protein levels are presented by fold-change calculated compared to WT-WD group. (f) PCoA analysis of brain metabolites (untargeted global metabolome) was conducted based on Bray-Curtis dissimilarity, representing the beta-diversity of each group, genotype, and diet. Significance was calculated using PERMANOVA with 999 random permutations. (g) The abundance of metabolites and the ranking of groups or diets by their average abundance are shown for brain metabolites. For the abundance heatmap, z-scores were calculated based on the average abundance of each group. Dendrograms were generated using hierarchical clustering results with the average linkage method, based on Bray-Curtis dissimilarity. (h) Spearman’s correlation results between diets within each genotype are shown for brain metabolites. See also Figure S9–10. *n*=8–9 per group (except for Western blot). Data are presented as mean ± SD. Significance was determined using unpaired t-tests between WT-WD and WT-MkD, as well as between AD-WD and AD-MkD. #p<0.1; *p<0.05; **p<0.01; ***p<0.001. WT: wild-type; AD: Alzheimer’s disease (APP/PS1 transgenic) mice; MkD: Mediterranean-ketogenic diet; WD: Western-style diet.

We next aimed to identify key metabolites derived from MkD that could contribute to reduced inflammation and improved barrier function, neurobehavior, and neuromuscular activity. To achieve this, we overlapped the metabolomic profiles of the feces and serum to screen the metabolites that could potentially be transported to the brain via the gut-brain axis. Lactate and leucine stood out as metabolites that were simultaneously more abundant in both the feces and serum following MkD, with lactate showing more pronounced differences in the feces than in serum while leucine displayed more distinct differences in serum compared to the feces based on diet (Figure 7a,b). To confirm that these metabolites are microbiota-derived, we executed the PICRUSt2 workflow to retrieve the functional readout of the microbiome. As anticipated, the comparative analysis of the relative abundance of genes associated with lactate or leucine production revealed a higher presence of genes involved in lactate production in MkD-fed mice, particularly genes encoding NAD^+^-dependent lactate dehydrogenase (*ldhA*), which is known to play a significant role in lactate production (Figure 7c). This corroborates that the MkD-modulated microbiome may have an increased capacity to produce lactate, which could be transported to the brain via circulation. On the other hand, the gut microbiome in WD-fed mice harbored more genes involved in leucine production (Figure S8A), suggesting the possibility that leucine may be derived from the MkD itself rather than being produced by the altered gut microbiota.

Additionally, to determine the potential role of lactate and leucine in mediating the favorable changes in tight-junction and inflammatory markers, as well as neurocognitive outcomes, we conducted correlational analyses (Figure S9A–D) wherein we observed an inverse correlation of intestinal lactate levels with hippocampal inflammatory markers IL-1β and IL-6. In addition, serum lactate levels showed negative correlations with brain inflammatory markers, including hippocampal IL-6, IL-8, and hypothalamic IL-10 (Figure S9A–B). Given that we found increased levels of these inflammatory markers to be associated with poor neurocognitive outcomes, it is worth noting that lactate levels in the feces were also negatively correlated with these outcomes (Figure S9C). Furthermore, fecal lactate exhibited a positive correlation with colonic CLDN12 and a negative correlation with colonic IL-17 (Figure S9A–B). The correlation between fecal and serum leucine with various markers was more pronounced compared to lactate. Fecal leucine displayed broader association arrays with markers of the intestine, while serum leucine showed stronger associations with markers of the brain. Commonly, both fecal and serum leucine exhibited negative correlations with colonic IL-8, hippocampal IL-1β, and IL-6, and positive correlations with ileum CLDN1 and colonic IL-10. Additionally, fecal leucine demonstrated a positive association with colonic CLDN12, while serum leucine was positively associated with hippocampal tight-junction proteins, including CLDN5 and ZO-1 (Figure S9C–D). Notably, hippocampal IL-1β and colonic CLDN12 showed significant correlations with motor coordination, and both fecal and serum leucine positively correlated with this outcome (Figure S9E).

To further confirm the brain-specific effects of diet and diet-induced changes in key metabolites, we quantified the levels of proteins involved in lactate metabolism and neurodegenerative disease pathology and assessed brain metabolomic profiles. Specifically, the expression levels of G-protein-coupled receptor 81 (GPR81), a lactate receptor distributed in the brain, tended to increase in MkD-fed mice and nearly reached significant augmentation in AD mice, although AD mice exhibited relatively lower expression compared to WT mice (Figure 7d,e). This finding suggests that the increased level of lactate following MkD enhances GPR81 expression in the brain. Subsequent brain metabolome analyses revealed distinct metabolite profiles between WD-fed and MkD-fed groups in AD mice (Figure 7f). AD-MkD mice showed higher levels of lactate and neurotransmitter-related metabolites such as aspartate, glutamate, and glutamine. Conversely, metabolic by-products like N-butyrylglycine and 3-Hydroxyisovalerate, along with energy metabolism-associated metabolites such as creatine and hypoxanthine, were more abundant in AD-WD mice. Notably, the metabolite profiles of WT mice differed significantly from those of AD mice. However, biosynthetic precursors like sn-Glycero-3-phosphocholine (GPC), a precursor of acetylcholine, and pyroglutamate were more abundant, while the acute neurotoxic byproduct succinylacetone decreased after MkD in both WT and AD mice (Figure 7g,h). Unlike fecal and serum metabolites, leucine was more abundant in WD-fed mice in both genotypes.

Furthermore, we measured specific hallmarks of AD, including Aβ and tau (total-tau and phosphorylated-tau), to assess the direct impact of the diet on pathogenesis. AD mice exhibited higher levels of Aβ compared to WT mice. Although Aβ levels decreased in WT-MkD mice, the change was not significant, and there was no difference observed in AD mice. Interestingly, total-tau levels varied among individual mice, but AD-WD mice tended to have higher levels compared to other groups. Regarding phosphorylated-tau, no significant differences were observed among the groups, although it was marginally decreased in the MkD-fed group (Figure 7d,e).

### MkD induces distinct transcriptomic changes in the hippocampus of AD mice

**Figure 8.**
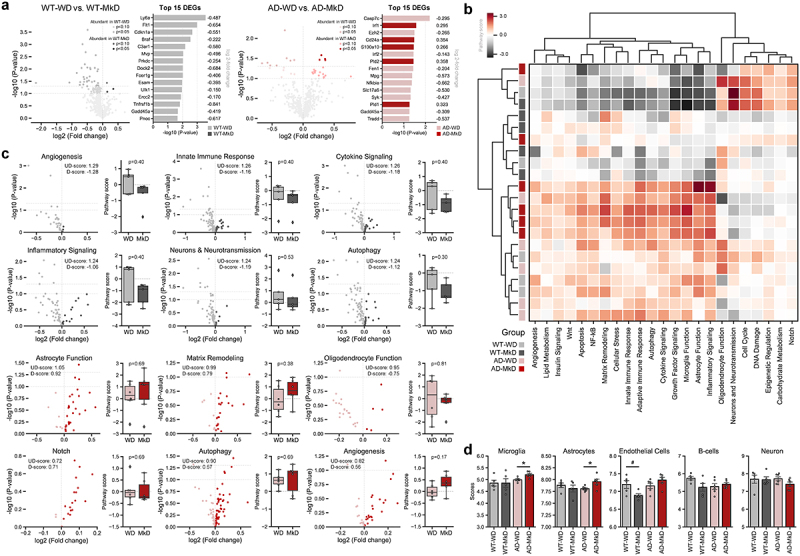
Mediterranean-ketogenic diet, versus standard Western-style diet, distinctly modulates the transcriptional arrays in hippocampus in APP/PS1 mice (a) volcano plots showing differentially expressed genes (DEGs) (log2 fold-change), and top 15 DEGs most significantly changed by diet. (b) Heatmap based on the pathway score of each sample for 23 neuroinflammation-associated pathways. The dendrogram was generated using hierarchical clustering results with the complete linkage method, based on Euclidean distance. (c) Volcano plots showing differentially expressed pathway associated DEGs and bar graphs of pathway signature scores of samples for top six differentially expressed pathways in WT and AD group respectively. (d) Predicted cell type abundance. Differences in pathway score and cell type were between WT-WD and WT-MkD and AD-WD, as well as AD-MkD were assessed with Mann–Whitney U test. *n*=6 per group. #p<0.1; *p<0.05. WT: wild-type; AD: Alzheimer’s disease (APP/PS1 transgenic) mice; MkD: Mediterranean-ketogenic diet; WD: Western-style diet.

Based on the findings of reduced pro-inflammatory cytokine expression in the hippocampal area and the predominance of GPR81 expression in the hippocampus, we next examined the transcriptional changes for 770 genes associated with 23 neuroinflammation pathways in the hippocampus using the NanoString Neuroinflammation panel. Overall, the profiles of differentially expressed genes (DEGs) were distinct between genotypes. WT mice exhibited a higher number of DEGs (34 genes; 1 upregulated, 33 downregulated) than AD mice (12 genes; 4 upregulated, 8 downregulated), with most genes showing downregulation in both groups (Figure 8a). Subsequently, pathway enrichment was assessed using a set of underlying genes. Interestingly, each WD-fed, WT-MkD, and AD-MkD mice clustered separately, indicating that mice within the same genotype-diet group shared similar pathway profiles (Figure 8b). AD-MkD mice demonstrated the most significant changes in pathway profiles, while WT-MkD mice showed the least changes compared to other groups (Figure 8b). More specifically, the top six most differentially expressed pathways (DEPs) based on global significance scores, including angiogenesis, innate immune response, and cytokine and inflammatory signaling, were all downregulated after MkD in WT mice. Meanwhile, among these six DEPs in AD mice, with the exception of the oligodendrocyte function, all other pathways, including astrocyte function, matrix remodeling, notch, autophagy, and angiogenesis, were upregulated after MkD (Figure 8c). Although these differences did not reach a statistically significant level and showed moderate variances in pathway signature scores, there was a clear trend that more genes associated with these pathways were highly expressed in one diet group compared to the counterpart. Additionally, we estimated the cell abundance using genes previously characterized for various cell populations. In the WT group, there were mostly no differences between diets, except for endothelial cells, which decreased nearly significantly in MkD-fed mice. Conversely, in the AD group, the abundance of microglia and astrocytes was higher in MkD-fed mice compared to WD-fed mice.

## Discussion

Nutritional and dietary modifications have emerged as promising strategies for the prevention and amelioration of neurodegenerative diseases, such as AD, by modulating the gut-brain axis. In this study, we conducted a comprehensive assessment of the ameliorative effect of MkD, a newly emerging dietary pattern, on AD neuropathology in a mouse model. Our findings revealed favorable outcomes in metabolic health, as well as improvement in neurocognitive and neuromuscular activities. These therapeutic outcomes were accompanied by specific shifts occurring in the gut microbial community, as well as gut-blood metabolites profiles.

Certain dietary patterns can disrupt systemic metabolic processes, thereby increasing susceptibility to AD.^23^ A WD pattern is known to contribute to metabolic disorders, including hepatosteatosis; and glucose homeostasis and insulin sensitivity are closely intertwined with the liver. Hepatic steatosis is prevalent among overweight individuals and is strongly associated with glucose intolerance and insulin resistance.^24^ Accumulation of fat in the liver diminishes hepatic insulin clearance, resulting in hyperinsulinemia-driven insulin resistance.^25^ In our study, mice subjected to WD exhibited increased liver weight, fat accumulation, and elevated levels of circulatory lipoproteins. Conversely, even though MkD-fed mice gained similar weight to those on the WD, they displayed lower liver weight and fat mass, suggesting that the MkD diet protected against hepatosteatosis, even without concurrent weight reduction, unlike WD.^26^ Also, our functional analyses revealed higher upregulation of insulin signaling pathways in WD-fed mice, as compared to MkD-fed mice, in both genotypes. This upregulation is in line with previous research involving obese mice with insulin resistance^27^ and demonstrates a causal association between the microbiome and insulin sensitivity.^28^ However, WD-fed mice did not exhibit signs of abnormal glucose intolerance or insulin resistance compared to MkD-fed mice. This discrepancy might be attributed to the relatively shorter duration of the study (12-weeks) compared to previous studies reporting impaired glucose/insulin homeostasis in WD-fed mice. For instance, a previous study examining the impact of hypertriglyceridemia on glucose homeostasis and insulin response also noted normal glucose metabolism and insulin sensitivity in mice during the early pathological stage (4 months) whereas, at a later stage (12 months), significant glucose intolerance and insulin resistance were recorded.^29^ It is plausible that the WD-induced impact on glucose/insulin regulation decreases insulin clearance ability during early pathological stages, which later leads to detectable impairment in glucose intolerance and insulin resistance at intermediate-late pathological stages during long-term intervention.

We found that the two dietary interventions distinctly shaped the gut microbiome and metabolomic profiles. Overall, the microbiome diversity tended to be increased after the MkD, and the microbiome composition differed significantly between MkD- vs. WD-fed mice. These findings are in line with previous studies by our team and others reporting the effects of MD or MkD interventions.^30–32^ Specifically, we found a profound proliferation of *Lactobacillus* in response to MkD, a signature similar to previous studies examining the microbiome in relation to MD or KD patterns,^30,33^ whereas WD reduced its abundance. Several *Lactobacillus* species and strains have been found to show beneficial effects on neurocognitive function by modulating the gut microbiota and the activity of enzymes related to AD pathology.^34,35^ These therapeutic effects of *Lactobacillus* partially originate from its metabolites. Notably, certain *Lactobacillus* spp. possess the glutamate decarboxylase (*gadB/gadC*) genes, enabling these strains to produce γ-Aminobutyric acid (GABA), an inhibitory neurotransmitter known to play a central role in prevention of neurological disorders by altering GABAergic circuits.^36^ Indigenous *Lactobacillus* strains have been found to harbor these genes and possess the ability to produce GABA.^37^ While GABA’s permeability through the blood-brain barrier (BBB) remains unclear,^38,39^ studies have shown that specific *Lactobacillus* intervention or modulation of the gut microbiota through a KD can increase GABA levels in the hippocampus.^40,41^ In our study, we observed a notable increase in glutamate, the precursor of GABA, and a higher functional abundance of glutamate decarboxylase gene (Figure S8B) in MkD-fed mice compared to the WD group. This finding may indicate that enhanced *Lactobacillus* may lead to increased production of GABA, thereby potentially exerting a protective impact on AD risk. Furthermore, all *Lactobacillus* species produce lactate through the action of lactate dehydrogenase.^42^ Our findings indicated that the MkD-fostered microbiota not only exhibited a significantly higher abundance of lactate dehydrogenase genes, but also comprised elevated levels of lactate in the feces and serum. Microbiota-derived lactate has been shown to have several positive effects on the host, including enhanced regulation of intestinal stem-cell-mediated epithelial development^43^ and proliferation of lactate-utilizing bacteria that metabolize lactate to butyrate.^44^ Additionally, lactate has been reported to regulate and enhance enteric immune and inflammatory responses by suppressing the toll-like receptor (TLR)-4 signaling pathway.^45^ The anti-inflammatory effects of lactate in the intestine have been confirmed for both dietary intake and lactate derived from the gut microbiota.^45,46^ Interestingly, we found a negative association between lactate levels and the expression of IL17 in the colon. Notably, TLR-4 regulates IL17 production and is required for IL17-induced tissue inflammation.^47^ Furthermore, we also found a significant co-occurrence pattern of lactate with taxa belonging to the Lachnospiraceae family, including the genus *Lachnoclostridium*. Species within the Lachnospiraceae family are known as lactate-utilizers and butyrate-producers.^48^ Likewise, *Lachnoclostridium* has been reported to possess similar functionality.^49^ Although we did not detect a favorable trend in SCFAs following MkD, these results might suggest that MkD shapes the gut ecological niche by altering microbiota composition, leading to concomitant increase in bacterial clades with a symbiotic relationship through their metabolites. Moreover, we explored the possibility of microbiota-produced lactate translocating to the brain and influencing neurological function. The well-established concept of the ‘gut-brain axis’ emphasizes bidirectional communication between these two organs mediated by several pathways involving bacterial fragments, neurotransmitters/neuropeptides, and microbial by-products and metabolites generated in the gut.^4^ In terms of bacterial metabolites, several of these are able to pass through or interact with the BBB as these travel through the circulatory system, thereby influencing the central nervous system (CNS) and its functions.^50^ GPR81, also known as Hydroxycarboxylic Acid Receptor 1 (HCAR1), a lactate receptor, which is enriched at the BBB and active in the mammalian brain,^51^ has been found to enhance cerebral vascular endothelial growth factor A (VEGFA), leading to increase in cerebral angiogenesis. Additionally, subcutaneous injection of L-lactate has been shown to increase lactate level in circulation, resulting in an augmentation of brain VEGFA and capillary density.^52^ AD has been inherently linked with ischemic brain condition, as infarction corresponds with Aβ deposition and reactive oxygen species (ROS) formation, both crucial factors in AD.^53^ In addition, it has been shown to play various neuroprotective roles by modulating energy metabolism and synaptic activity.^54^ In our findings, along with an elevated lactate level in the serum, GPR81 increased after MkD, particularly in AD mice, and angiogenesis pathway was mildly upregulated in the hippocampal area. Furthermore, lactate administration ameliorated LPS-induced neuroinflammation by blocking the transport function of monocarboxylate transporter 1 (MCT1) in microglia, leading to improvements in sickness behavior, such as enhancements in total distance in the OFT and performance on the rotarod.^55^ This finding was further validated by significant negative correlations of fecal and serum lactate levels with inflammation markers in the hippocampus, as well as positive correlations with the outcomes of behavioral assays. Particularly noteworthy, both IL-1β and IL-6 exhibited negative correlations; these interleukins are typically stimulated by LPS, but their levels are found to be reduced in MCT1 knockdown cells.^55^ Also, IL-6 has been linked with cognitive impairments, and the neutralization of elevated IL-6 in the brains of AD mice has been found to alleviate memory impairment.^56^ Similarly, dietary lactate treatment has been shown to improve memory function by increasing GABA level in the hippocampus.^57^ Collectively, these findings indicate a mechanism through which (MkD-fostered) microbiota-derived lactate may be transported to the brain via the gut-blood-brain channel, thereby contributing to the mitigation of AD-associated neuroinflammation and neurocognitive impairment through its interaction with transporters and receptors, including GPR81.

An important aspect to consider is the surge in AAs that accompanies the host adaptation to the MkD pattern. The increase in AAs mostly results from a KD, whereas traditional MD contains fewer AAs than WD.^11^ Among the elevated AA arrays following MkD, we found leucine to be the most substantially and significantly increased AA in both serum and feces levels. Leucine is known to play a pivotal role in maintaining intestinal integrity and function through the activation of the mammalian target of rapamycin (mTOR) pathway, which stimulates protein synthesis in intestinal epithelial cells.^58^ Our findings revealed several favorable correlations of fecal leucine levels with markers of tight-junction integrity and inflammation in both the ileum and colon, possibly arising from improved gut epithelial integrity. Furthermore, leucine is known to readily and rapidly cross the BBB via the solute-carrier transporter 7a5 (SLC7A5; also known as LAT1), undergoing rapid transamination to the production of neurotransmitters or their precursors, such as glutamate and ketoleucine.^59^ Astrocytes, particularly situated in close proximity to brain capillaries, serve as the initial metabolic site for leucine.^60^ Leucine accounts for up to 25% of the total glutamate nitrogen content, serving as a significant contributor to brain glutamate.^61^ Given its status as an essential AA, dysregulation or impairment in leucine transporters can trigger neurological and neurobehavioral abnormalities, increasing the risk of AD.^59,62^ Our findings revealed an increased abundance and enhanced function of astrocytes in the hippocampus, suggesting that leucine might more actively convert to neurotransmitters, especially in AD mice. However, the brain’s leucine level was relatively lower in MkD, presumably due to rapid uptake and transamination in astrocytes.^63^ Moreover, our findings indicated a negative correlation of serum leucine levels with hippocampal IL-6 and IL-1β as well as hypothalamic TNF-α. We also noticed a positive correlation of serum leucine with CLDN5, which is highly enriched in BBB and linked to neurodegenerative disorders,^64^ and a similar pattern with ZO-1. However, the quantification of both proteins showed reverse trends compared to mRNA transcripts, suggesting potential discrepancies between transcription and resulting protein production. Given the possibility of several influencing factors such as steady state, signal delay, and post-transcriptional processes, more studies focusing on tight-junction proteins in the brain are necessary.^65^ Additionally, leucine levels showed a positive correlation with performance in the hanging-wire neuromuscular coordination test. Administration of leucine-enriched essential AAs has previously been shown to reduce IL-6 expression in stressed muscles and promote muscle recovery.^66^ Clinically, leucine supplementation has been found to attenuate C-reactive protein, an inflammatory marker that increases following IL-6 secretion, thereby improving muscle strength in patients with cerebral palsy.^67^ Although these studies did not assess the inflammatory profiles within the CNS, these do demonstrate a connection between IL-6 and neuromuscular function, underscoring the ameliorative effect of leucine. Concomitantly, BCAAs supplementation has been reported to reduce hippocampal IL-1β levels, which is exacerbated by obesity.^68^ Conversely, studies have also reported specific adverse effects of elevated BCAA levels, including leucine, on obesity, insulin resistance, and metabolic health^69,70^; however, studies have suggested that such negative outcomes are more likely linked to the relative quantity of dietary BCAAs rather than the elevated BCAAs themselves.^71^

We also found a noteworthy increase in the abundance of *Akkermansia*, *Lachnoclostridium*, *Parasutterella*, and *Bacteroides* in response to MkD. These findings align with previous research demonstrating a reproducible increase in these genera through an MD.^72^ A growing body of evidence underscores the pivotal role of *Akkermansia muciniphila*, a mucin-degrading bacterium, in influencing the host brain function via the gut-brain axis. Studies actively investigating its potential role in AD have proposed several plausible mechanisms, including regulation and protection of the integrity of the intestinal mucosal barrier and modulation of immune responses and metabolites.^73^ *Parasutterella*, a core bacterial member of the human and murine gut microbiome, may significantly contribute to host metabolic functions by regulating the bile acid (BA) profiles via influencing the expression of ileal BA transporter genes and hepatic BA synthesis genes. Our findings revealed a substantial reduction in BAs in both genotypes of mice following MkD, which is consistent with earlier findings from Mediterranean-style dietary interventions.^74,75^ While certain BAs play a pivotal role in shaping the microbiome composition and metabolic activities, an excessive production or imbalance between primary and secondary BAs can lead to gut dysbiosis and DNA damage due to their inherent toxicity. Imbalances in BAs have been implicated in conditions such as recurrent *Clostridioides difficile* infection and inflammatory bowel disease.^76^ In this context, our findings suggest that MkD may contribute to the maintenance of gut and metabolic health by efficiently reducing BAs in the gut through the proliferation of *Parasutterella* and a reduction in dietary cholesterol, which serves as a precursor to BA.

Regarding the various changes observed in the brain, including alterations in protein expression, metabolites, and inflammatory pathway-associated gene transcription levels, both genotypes exhibited distinct changes after MkD. These differences likely arise from alternations in transcriptional networks commonly found in transgenic models expressing mutant forms of the amyloid precursor protein.^77^ Notably, AD mice have over 2-fold more Aβ than WT mice, indicating the effect of gene manipulation on AD mice, elevating Aβ production while disrupting transcriptional networks. Despite this, there was an increase in GPC in MkD-fed mice in both genotypes, particularly pronounced in AD mice. GPC, a naturally occurring choline compound in the brain, serves as a precursor to acetylcholine. Acetylcholine, the primary neurotransmitter implicated in learning and memory, has been investigated for treating dementias, including AD.^78,79^ Moreover, succinylacetone, a byproduct of tyrosine degradation, decreased in both genotypes following MkD. Accumulation of succinylacetone is known to inhibit neurotransmission by increasing aminolevulinic acid, inducing the formation of free radicals, and causing oxidative damage.^80^ Furthermore, the abundance of microglia and astrocytes notably increased in AD-MkD mice. Although both cells play crucial roles in brain health and function, they have ambivalent roles in neuroinflammation and neurodegeneration. Reactive astrocytes are divided into two phenotypes: A1 (neurotoxic) and A2 (neuroprotective). Similarly, activated microglia consist of M1 (pro-inflammatory) and M2 (anti-inflammatory) phenotypes.^81,82^ Although we could not examine whether the increased astrocytes and microglia are in a reactive state, they may not represent the A1 or M1 phenotype. This assumption is supported by the significant downregulation of the Caspase 7 gene (Casp7c), which activates microglia driving neurodegeneration, and TNF-α, forming a positive feedback loop with activated microglia. In contrast, the S100 calcium-binding protein A10 (S100a10), identified as a specific marker of A2-astrocytes, was upregulated in AD-MkD mice compared to AD-WD mice.^83–85^ In terms of AD biomarkers, although there were no changes observed in Aβ and phosphorylated-tau levels following MkD, the total-tau level was higher in AD-WD mice compared to all other groups, whereas the level of total-tau in AD-MkD mice was nearly identical to that in WT mice. Both total-tau and phosphorylated-tau are known to increase in AD, with total-tau levels particularly associated with alterations in neuronal plasticity and BBB dysfunction.^86,87^ One potential mechanism behind this dysfunction is the upregulation of the autophagy pathway. Our transcriptional analysis revealed an upregulation of the autophagy-associated pathway following MkD in the AD group. Previous studies have reported that the tau protein can be degraded via the autophagy-lysosome system, and enhancing autophagy function has been proposed as a promising therapeutic strategy for reducing tau accumulation.^88,89^ Further studies are needed to better evaluate how MkD upregulates autophagy.

The current study has limitations that should be noted when interpreting our results. Firstly, we used female mice based on prior studies reporting more consistent and reproducible patterns in the appearance of neuropathological symptoms in females versus males in this model. Given the sexual dimorphism in AD predisposition, as well as in microbiome composition, our study could not assess sex-specific mechanisms that may differ in male models. Second, our intervention duration of 12 weeks was chosen to capture assessments during the intermediate pathological trajectory in order to evaluate the preventative potential of MkD. However, this duration results in nearly identical performance in behavioral assays between WT and AD mice. Given that AD in humans is typically detected at age 65 years or older, future studies should examine the efficacy of MkD in aging models in the absence of genetic predisposition. Although we used WD and MkD diets to mimic the composition of human diets, the effects of diet can vary in humans due to differences in the microbiome and metabolism between humans and mice.^90,91^ Previous transcriptome profiles have shown significant differences in neurotransmitter receptors and ion channels, which play a major role in brain function between humans and mice.^92^ Consequently, sometimes opposite conclusions are drawn regarding similar dietary interventions for humans and mice.^93^ Therefore, to precisely understand the effects of MkD on human AD pathology, long-term clinical intervention studies focusing on dietary effects are necessary. Nevertheless, the study has several strengths and advantages. To our knowledge, this is the first comprehensive study executing multi-omics approaches and assessing multi-organ elements to systematically capture entero-neuro-physiologic responses to an innovative and clinically-translational dietary pattern in a validated preclinical AD model. Taken together, our findings demonstrate that MkD pattern could offer advantageous dietary components, including leucine, while specifically modulating the gut microbiome-metabolome arrays characterized by sequentially fostered population of lactate-producing, lactate-to-butyrate metabolizing and butyrate-producing taxa including *Lactobacillus*, *Lachnoclostridium, Parasutterella*, and *Bacteroides*. This leads to elevated arrays of microbiota-derived metabolites viz. lactate and butyrate across the gut-blood-brain niches, eventually improving the AD-related neurocognitive and neuromotor function. Notably, some of these outcomes manifest favorable effects in both transgenic AD mice and wild-type counterparts, indicating that MkD-induced mechanisms may prevent metabolic disorders and delay AD-like pathology independent of genetic predisposition. The findings corroborate the emerging evidence underscoring the role of nutritional elements and diet-microbiome interactions in AD pathology. Further, the study reveals potential microbiome-associated mechanisms through which Mediterranean, ketogenic, or the newly invented MkD patterns may regulate intestinal, metabolic, and neurocognitive health in individuals at-risk for AD or aging-associated metabolic disorders.

## Materials and methods

### Animal studies

The overall experimental design is summarized in Figure 1A. Female APP/PS1 double-transgenic (AD) mice (B6.Cg‐Tg(APPswe, PSEN1dE9)85Dbo/Mmjax; on C57BL/6J background; *n* = 18) and wild-type (WT) littermates (*n* = 18) were obtained from the Jackson Laboratory Inc. (Bar Harbor, ME, USA) at the age of 6-weeks. Mice were housed in individual cages and were given a two-week period to acclimatize to the new vivarium conditions. Both sets of mice were then randomly divided into two groups using simple randomization (*n* = 9 per group) and were housed in individual cages until the end of experiment. These groups were provided with an *ad libitum* approach to feeding, receiving a WD or the MkD for a total duration of 12 weeks. All the diet formulations had the same caloric content (details in Supplementary Table S1). Fecal samples were collected at the beginning of the intervention, at 4-weeks, and at 8-weeks post-intervention. Mice were placed in sterile 1 L polypropylene beakers until they passed at least 2–3 fecal pellets. These fecal samples were immediately preserved at − 80°C for further analysis. Following the 8-week nutritional intervention, the mice underwent a series of assessments as outlined in the subsequent sections. Weekly measurements were taken for bodyweight and diet intake. Upon reaching the end (12-weeks of intervention, at age 20-week) of the intervention period, the mice were anesthetized using isoflurane and then humanely euthanized by cervical dislocation. Measurements were taken for cecum weight, liver weight, and total gastrointestinal length. Tissues were collected, snap-frozen in liquid nitrogen, and were immediately preserved at − 80°C for further analysis. All animal studies and protocols were approved by Florida State University’s Institutional Animal Care and Use Committee (Protocol #202100008).

### Diet

The dietary patterns WD (with a calorie distribution of 35% fat, 50% carbohydrate, 15% protein) and MkD (consisting of 66% fat, 10% carbohydrate, 24% protein) were developed in collaboration with Research Diets Inc. (New Brunswick, NJ, USA). We closely emulated the composition of human diets, including comparable macronutrient ratios, fiber content, ingredient types, and fatty acid proportions, aligning with common human eating habits.^94^ The WD (Product #D21080102) regimen incorporated essential components like sucrose, corn oil, anhydrous butter, and cooked beef. Its formulation drew inspiration from the 2008 Dietary Assessment of Major Food Trends conducted by the US Department of Agriculture. In contrast, the MkD (Product #D21080103) pattern predominantly included ingredients such as wheat starch, Inulin fiber, olive oil, Menhaden oil, flaxseed oil, egg white, and fish protein isolate. These components match the MkD model used in our previous clinical investigations.^18,19^ Both dietary plans were designed in a way to maintain an equivalent caloric value (~3.65 kcal/g). Mice had unrestricted access to water throughout the duration of study.

### Body-composition

At the study endpoint, total body-composition including lean mass, fat mass, and total water was measured in live mice by using the EchoMRI-130 Body-Composition Analyzer (EchoMRI, MRI that Counts, Houston, TX, USA).

### Glucose and insulin tolerance tests

Glucose tolerance test (GTT) and insulin tolerance test (ITT) were completed as described in our previous reports.^95^ Tests were conducted subsequent to the collection of the final time-point fecal samples and all behavioral assays. Briefly, for GTT, the mice were fasted for six hours whereafter a glucose solution (2.5 g/kg bodyweight) was orally administered to the mice, and their blood glucose levels were measured at 0 minutes (before the injection), as well as at 15, 30, 60, and 120 minutes after the glucose administration. For ITT, the mice were fasted for four hours, and insulin (Humulin) was injected intraperitoneally. The mice’s blood glucose levels were assessed at 0 minutes (before the injection), as well as at 15, 30, 60, and 120 minutes after the injection.

### In-vivo gut permeability

Gut epithelial permeability was measured using the fluorescein isothiocyanate (FITC)-dextran assay as per our previously described method.^95^ Tests were conducted subsequent to the collection of the final time-point fecal samples and all behavioral assays. Briefly, the mice were fasted for 4 hours prior to the experiment and were orally administered a solution of fluorescein isothiocyanate (FITC) dextran (4 kDa) at a dose of 60 mg/100 g bodyweight. Two-hours after administration, while still on fast, 100 μL of blood was collected from a tail vein using a heparinized capillary tube. The serum concentration of FITC-dextran was measured using a fluorescence plate reader at 530 nm with excitation at 485 nm.

### Gut microbiome analysis

Gut bacterial and fungal microbiome profiles were measured and analyzed as per our previously described methods.^96^ The fecal samples were preserved at −80°C until microbial DNA extraction. The QIAmp PowerFecal Pro DNA Kit (Qiagen) was used to extract genomic DNA from a 200 mg fecal specimen, following the manufacturer’s guidelines. Universal primers 515F (barcoded) and 806 R were employed to amplify the hypervariable V4 region of the bacterial 16S rRNA gene and ITS1f and ITS2 (barcoded) were used to amplify the internal transcribed spacer (ITS) region of the fungal rRNA gene, in accordance with the Earth Microbiome Project benchmark protocol (http://www.earthmicrobiome.org). Subsequently, the resulting amplicons were purified using AMPure® magnetic purification beads (Agencourt) and quantified by using a Qubit-4 fluorometer (InVitrogen). Equal molar concentrations of the libraries were pooled into one and the final amplicon library was sequenced for paired-end (2 × 300bp) sequencing using an Illumina MiSeq sequencer (using Miseq reagent kit v3; Illumina Inc., San Diego, United States).

### Metabolomics analysis

Fecal, blood, and brain metabolomic arrays were measured by executing the global untargeted approach through the use of a high-throughput Nuclear magnetic resonance(NMR) system, as described in our previous reports.^97^ Fecal, serum, and brain samples from the mice were processed following the previously described protocol^98^ with minor modifications. Samples were extracted through vortexing for five minutes with deionized water. The extracted samples were combined with a phosphate buffer (pH = 7.4) in D2O to create a final solution containing 10% D2O, 0.1 M c, and 0.1 mM Trimethylsilyl propionate (TSP). Following centrifugation, the samples were transferred to 5 mm NMR tubes and analyzed using a Bruker Ascend 400 MHz high-resolution NMR system (Bruker Biospin, Germany). For all samples, a 1D first increment of a NOESY (noesygppr1d) experiment with water suppression was conducted, employing 64 scans. Subsequent NMR processing included phasing and referencing to TSP in TopSpin 4.06 (Bruker BioSpin, Germany). NMR spectra were processed in Amix 4.0 (Bruker BioSpin), and automated binning was applied, as previously described,^99^ to minimize peak overlap and splitting. Metabolite quantification was performed using Chenomx 8.6 (Chenomx Inc). Total intensity normalization was applied prior to further data analysis.

### Neurocognitive and neuromuscular function

#### Open-field test

The examination was conducted in accordance with a previously established protocol.^100^ The assessment took place in a designated room within the vivarium facility to evaluate fundamental locomotor activity. Mice were carefully positioned at the center of a sanitized chamber measuring 40 × 40 cm^2^, and their movements were recorded via video for a duration of 5 minutes. Behavior parameters, including mobility, total distance covered, and the percentage of time spent in the central area, were analyzed using the Ethovision XT software (Noldus).

#### Novel object test

The examination was conducted following a previously described protocol,^101^ omitting a preliminary habituation phase. A pair of similar objects were positioned within the same chamber used for the open field test, enabling the mice to explore them for 5 minutes. After 24 hours, the mice underwent a test to evaluate memory retention. This test was achieved by placing two objects in the chamber, keeping their original positions while replacing the second object (a familiar wooden cube) with a third object (a novel black rubber cylinder). The orientation of the mice was once again monitored for 5-minutes. The Ethovision XT software was utilized to measure the amount of time the mice spent exploring the objects, specifically when their nose tip came within a radius of 2 cm from the objects. Memory retention was calculated using a discrimination index formula: (time spent exploring the objects at their original location − time spent exploring objects at the new location)/total exploring time for all objects.

#### Location memory test

The examination was conducted in accordance with a previously established protocol^101^ with some modifications. Four distinct objects were evenly positioned within the previously described chamber. The mouse was gently situated at one end, facing the arena wall, and was permitted to explore freely for a duration of 5 minutes. After a 24-hour interval, the positions of two adjacent objects were interchanged, and the mouse’s retention of spatial memory was evaluated through a 5-minute video recording of its behavior. The Ethovision XT software was used to ascertain the time the mouse spent exploring the objects, registering instances when the mouse directed its nose tip within a 2 cm radius of the objects. The calculation of memory retention utilized a discrimination index formula: (time spent exploring the relocated objects − time spent exploring the familiar object)/total exploring time.

#### T-maze spontaneous test

The spatial working memory of rodents was assessed using this test, following the previously described protocol.^102^ The mice were gently placed at the far end of the initial arm, with their heads facing the southern wall. Subsequently, the mice were allowed to move freely, and any alterations they made (either left or right) in the goal arms were recorded. Each mouse underwent seven trials, and the percentage alteration score, which acts as an indicator of working memory, was calculated using the formula: (total number of correct alternations/6) * 100.

#### Neuromuscular and motor function test

The neurobehavioral coordination of the mice was evaluated using grip strength, rotarod, and hanging wire tests. To measure forelimb grip strength, a grip strength meter (World Precision Instruments) was employed. Each mouse’s forelimbs were placed on a brass pole, and its tail was gently pulled until its grip was released. This procedure was conducted for three trials per mouse. During the rotarod test, the mice underwent a 5-minute training session in which they were positioned on a Rotarod apparatus (Harvard Apparatus) rotating at a consistent speed of 4 rpm. On a subsequent day, the mice were tested by placing them on the apparatus and initiating rotation at 4 rpm, with speed increments of 1 rpm every 8 seconds. The time taken for each mouse to fall from the rotarod was recorded, and each trial, accompanied by a 5-minute rest interval, was conducted three times for each mouse. The hanging wire test was executed following the outline provided by Hoffman et al. .^103^ In essence, a circular wire (32 cm long and 2.5 mm in diameter) was securely fastened on a stand, enabling free circular motion. The mice were positioned beneath the wire, ensuring that all four paws were in contact. Three distinct hanging times were recorded, with a 30-second interval between each measurement.

### Gene expression analyses

Snap-frozen intestinal (colon and ileum) and brain (hippocampus and hypothalamus) were used to extract total RNA using the RNeasy kit (Qiagen). Subsequently, the High-capacity cDNA reverse transcription kit (ThermoFisher) was used for reverse transcription. The quantification of mRNA expression for tight junction proteins and inflammatory markers were quantified using real-time PCR (QuantStudio3, Applied Biosystems) with primers listed in Supplementary Table S2. For internal normalization, the 18S gene was employed as a housekeeping control. The results were expressed as ddCt method, normalized against the 18S expression in the WT-WD group.

### Western blot

Before the experiment, all the primary antibodies (all purchased from Invitrogen, Waltham, MA, USA), including IL-1β (cat# P420B), IL-6 (cat# P620), TNF-α (cat# AMC3012), claudin-5 (cat# 35–2500) zonulin-1 (cat# 40–2200), GPR81 (cat# PA5–114741), Aβ (cat# 51–2700), total-tau (cat# 13–6400), phosphorylated-tau Ser396 (cat# 44-752 G), and beta-actin (cat# MA5–15739), were diluted 1:1000 in a 3% Bovine Serum Albumin buffer with 0.1% azide. Homogenized brain tissues were lysed using Buffer A (50 mM HEPES, pH 7.4, 150 mM NaCl, 1 mM EGTA and 0.1 mM MgCl_2_) containing 1% Triton X-100, 2 mM sodium orthovanadate, 10 mM sodium pyrophosphate, 10 mM sodium fluoride, and protease inhibitor cocktail (Sigma, P340-5 ML) for 20 minutes on ice. After 1 minute of centrifugation, only the supernatant was transferred into a new tube and was subjected to sonication using a QSonica sonicator for 10 seconds. The protein content was quantified at 595 nm using the Bradford Assay in a spectrophotometer. Total extracts (50 µg) were separated in 4–20% acrylamide precast Criterion gels (BioRad, Hercules, CA, USA). The gels were transferred onto a PVDF membrane utilizing the Owl HEP-1 semidry system (Thermo Scientific, Waltham, MA, USA). Subsequently, the membranes underwent a 20-minute blocking phase in TBS (150 mM NaCl, 2 mM KCl, 25 mM Tris, pH 7.4) containing 1.5% nonfat dry milk (Bio-Rad, Hercules, CA, USA). Following a wash in TBS, the membranes were exposed to primary antibodies for a duration of 1 to 2 hours or overnight. Next, the membranes were rinsed three times in TBS-T (TBS with 0.05% T-X100) for 10 minutes each and were then subjected to a 45-minute incubation with HRP-conjugated secondary antibodies in a blocking buffer. After three TBS-T washes, the membranes were developed employing Pierce ECL Western blotting substrate (Thermo Fisher Scientific, Waltham, MA, USA).

### Nanostring neuroinflammation transcriptional analysis

One hundred ng of total hippocampal RNA underwent analysis using the NanoString nCounter® system, specifically the Mouse Neuroinflammation Panel (NanoString Technologies, Seattle, WA) to assess RNA transcript counts for 770 genes associated with 23 neuroinflammation pathways along with 13 internal reference genes. The counts of gene transcription were adjusted by the geometric mean of internal reference genes before subjecting them to detailed analysis using nSolver analysis software (ver. 4.0). The pathway signature scores, calculated from nSolver advanced analysis, were employed to summarize alterations in the expression levels of groups of genes associated with specific biological pathways. These pathway scores were generated through the first principal component analysis score of each sample, based on its gene expression levels across a particular pathway’s measured genes. Consequently, a positive score would signify an abundance of upregulated genes within the pathway, while a negative score would indicate a prevalence of downregulated genes. In addition, global significance statistics quantify the degree of differential expression among the genes within a gene set concerning a covariate, irrespective of whether individual genes are upregulated or downregulated. On the other hand, directed global significance statistics gauge how much the genes within a gene set are either upregulated or downregulated concerning the variable. Cell type abundance was assessed using genes that have previously demonstrated characteristic traits of different cell populations, serving as markers to quantify the abundance of these specific populations. Differences in pathway score and cell type were assessed with Mann – Whitney U test.

### Bioinformatics and statistical analysis

The microbiome sequencing data was processed using QIIME2 (ver. 2–2023.5).^104^ Raw sequences were demultiplexed and filtered based on their quality using the q2-demux plugin, followed by trimming and denoising through DADA2.^105^ All identified amplicon sequence variants (ASVs) were aligned utilizing MAFFT.^106^ Taxonomy assignment for the ASVs was carried out using the sklearn classifier with the pre-trained naïve Bayes taxonomy classifier, aligned against the 99% SILVA 138 database (bacterial taxonomy) or the 99% UNITE 9.0 database (fungal taxonomy). Chao1 (richness) and the Shannon index (richness and evenness) were employed as measures of alpha diversity. For beta-diversity analysis, the Bray-Curtis dissimilarity index was used, and these results were visualized through principal coordinate analysis (PCoA). Non-parametric Kruskal-Wallis test and PERMANOVA with 999 random permutations were applied to identify significant differences in microbial diversity and structure. Metagenomic functional activities were predicted using the open-source bioinformatics tool Phylogenetic Investigation of Communities by Reconstruction of Unobserved States 2 (PICRUSt2).^107^ Sequences were input into PICRUSt2 to predict the functional genes of classified members within the gut microbiota. The inferred gene families were subsequently annotated against Kyoto Encyclopedia of Genes and Genomes (KEGG) orthologs and grouped into KEGG pathways to generate functional pathways. Differential abundance of taxa, metabolites, and predicted functional pathways were identified using methods such as Linear Discriminant Analysis (LDA) effect size (LEfSe),^108^ the ANOVA-Like Differential Expression (ALDEx2)^109^ approach, and Spearman’s correlation. To predict the group using microbiome and metabolite patterns through supervised classification, the q2-sample-classifier plugin in QIIME2 was employed. A nested stratified 5-fold cross-validation was performed using the Random Forest classifier grown with 5,000 trees. To determine taxa that consistently increased or decreased over time, Spearman’s correlation was used. To assess the impact of two factors, genotype and diet, on taxa abundance and potential interaction effects, a two-way ANOVA was conducted. Visualization utilized ‘R’ or ‘Python’ packages.

## Supplementary Material

Supplemental Material

## Data Availability

The data that support the findings of this study are openly available in National Center for Biotechnology Information (NCBI)-Sequence Read Archive (SRA) at https://www.ncbi.nlm.nih.gov/sra, reference number PRJNA1011002 (16S rRNA sequencing dataset) and PRJNA1010986 (ITS rRNA sequencing dataset).
