# Supplementary material for: A modified Mediterranean-style diet enhances brain function via specific gut-microbiome-brain mechanisms
Source: Gut Microbes. 2024 Mar 6;16(1):2323752. doi: 10.1080/19490976.2024.2323752 (PMC10936641; doi:10.1080/19490976.2024.2323752)
Supplement: Supplemental Material [file KGMI_A_2323752_SM1749.zip › Supplementary table S2.docx]

**Table S2. Primer sequences**

| Primer | Sequence | Source |
| --- | --- | --- |
| Universal primer for V4 region of bacterial 16S rRNA gene | 515F-GTGYCAGCMGCCGCGGTAA,  806R-GGACTACNVGGGTWTCTAAT | https://earthmicrobiome.org/protocols-and-standards/16s/ |
| Universal primer for fungal ITS rRNA gene | ITS1f-CTTGGTCATTTAGAGGAAGTAA,  ITS2-GCTGCGTTCTTCATCGATGC | https://earthmicrobiome.org/protocols-and-standards/its/ |
| 18S | F-AGAAACGGCTACCACATCCA,  R-CCCTCCAATGGATCCTCGTT | Miranda-Ribera et al.^1^ |
| Claudin1 | F-GGCTTCTCTGGGATGGATCG, R-CTTTGCGAAACGCAGGACAT | Miranda-Ribera et al.^1^ |
| Claudin3 | F-CCTAGGAACTGTCCAAGCCG, R-CCCGTTTCATGGTTTGCCTG | Miranda-Ribera et al.^1^ |
| Claudin5 | F-GTTAAGGCACGGGTAGCACT, R-TACTTCTGTGACACCGGCAC | Miranda-Ribera et al.^1^ |
| Claudin12 | F-GAGCCGATGTGCTCCTGTT, R-GGAGGGCTTGAGCTGTATGG | Miranda-Ribera et al.^1^ |
| NO1 | F-AAGAAAAAGAATGCACAGAGTTGTT, R-GAAATCGTGCTGATGTGCCA | Miranda-Ribera et al.^1^ |
| Occldn | F-CTGACTATGCGGAAAGAGTTGAC, R-CCAGAGGTGTTGACTTATAGAAAGAC | Miranda-Ribera et al.^1^ |
| IL-1β | F-GAAATGCCACCTTTTGACAGTG, R-TGGATGCTCTCATCAGGACAG | Hu et al.^2^ |
| IL-6 | F-GTCCTTCCTACCCCAATTTCCA, R-CGCACTAGGTTTGCCGAGTA | Miranda-Ribera et al.^1^ |
| IL-8 | F-ACTCAAGAATGGTCGCGAGG, R-GTGCCATCAGAGCAGTCTGT | Miranda-Ribera et al.^1^ |
| IL-10 | F-TGGGTTGCCAAGCCTTATCG, R-TTCAGCTTCTCACCCAGGGA | Miranda-Ribera et al.^1^ |
| IL-17 | F-TTTAACTCCCTTGGCGCAAAA, R-CTTTCCCTCCGCATTGACAC | Miranda-Ribera et al.^1^ |
| TNF-α | F-GATCGGTCCCCAAAGGGATG, R-TTTGCTACGACGTGGGCTAC | Miranda-Ribera et al.^1^ |

1. Miranda-Ribera A, Ennamorati M, Serena G, Cetinbas M, Lan J, Sadreyev RI, Jain N, Fasano A, Fiorentino M. Exploiting the zonulin mouse model to establish the role of primary impaired gut barrier function on microbiota composition and immune profiles. Frontiers in Immunology 2019; 10:2233.

2. Hu Y, Fryatt GL, Ghorbani M, Obst J, Menassa DA, Martin-Estebane M, Muntslag TA, Olmos-Alonso A, Guerrero-Carrasco M, Thomas D. Replicative senescence dictates the emergence of disease-associated microglia and contributes to Aβ pathology. Cell reports 2021; 35.
