# Supplementary material for: A modified Mediterranean-style diet enhances brain function via specific gut-microbiome-brain mechanisms
Source: Gut Microbes. 2024 Mar 6;16(1):2323752. doi: 10.1080/19490976.2024.2323752 (PMC10936641; doi:10.1080/19490976.2024.2323752)
Supplement: Supplemental Material [file KGMI_A_2323752_SM1749.zip › Supplementary table S1.docx]

**Table S1. Detailed nutritional contents and components of Mediterranean-ketogenic diet and standard Western-style diet.**

| **Product #** | **WD (#D21080102)** | | | **MkD (#D21080103)** | | |
| --- | --- | --- | --- | --- | --- | --- |
| **dietary components** | **g** | **%** | **kcal (%)** | **g** | **%** | **kcal (%)** |
| Carbohydrate | 455.3 | 52.6 | 1821 (50) | 91.6 | 12.8 | 367 (10) |
| Protein | 136.9 | 15.8 | 547 (15) | 217.4 | 30.4 | 870 (24) |
| Fat | 143.2 | 16.6 | 1289 (35) | 272.3 | 38 | 2451 (66) |
| Cholesterol | 1.67 | 0.194 |  | 0.15 | 0.021 |  |
| Fiber | 22.7 | 2.6 |  | 50.8 | 7.1 |  |
| Total |  |  | 3658 |  |  | 3687 |
| **Fat contents** | **g** | **%** | **kcal %** | **g** | **%** | **kcal %** |
| SFA | 37 | 4.3 | 9.1 | 23.2 | 3.2 | 5.7 |
|  | 35.6 | 4.1 | 8.8 | 81.9 | 11.4 | 20 |
| PUFA | 6.2 | 0.7 | 1.5 | 20.6 | 2.9 | 5 |
| Total n-6 | 4.9 | 0.6 | 1.2 | 14.7 | 2.1 | 3.6 |
| Total n-3 | 1.2 | 0.1 | 0.3 | 5 | 0.7 | 1.2 |
| n-6/n-3 Ratio | 3.9 | | | 2.9 | | |
| 12+14+16 | 24.5 | 2.8 | 6 | 18.1 | 2.5 | 4.4 |
| Oleic | 33.1 | 3.8 | 8.1 | 78.6 | 11 | 19.2 |
| Linoleic | 4.8 | 0.6 | 1.2 | 14.3 | 2 | 3.5 |
| **Contribution to Protein (gm %)** | | | | | | |
| Casein | 24.5 | | | 24 | | |
| Fish Protein Isolate | 5.9 | | | 39.3 | | |
| Egg White | 32.6 | | | 9.3 | | |
| Beef, Cooked | 34.8 | | | 26 | | |
| L-Cystine | 2.2 | | | 1.4 | | |
| **Contribution to Carbohydrate (gm %)** | | | | | | |
| Corn Starch | 5.9 | | | 0 | | |
| Maltodextrin | 0 | | | 0 | | |
| Wheat Starch | 38.4 | | | 76.8 | | |
| Potato Starch | 5.3 | | | 0 | | |
| Sucrose | 45.6 | | | 23.2 | | |
| Fructose | 4.9 | | | 0 | | |
| **Contents (g)** | | | | | | |
| Casein | 38.5 | | | 60 | | |
| Fish Protein Isolate | 8.5 | | | 90 | | |
| Egg White | 55 | | | 25 | | |
| Beef, Cooked, Powdered, 5013 | 76.9 | | | 91 | | |
| L-Cystine | 3 | | | 3 | | |
| Corn Starch | 30 | | | 0 | | |
| Wheat Starch | 195 | | | 75 | | |
| Potato Starch | 30 | | | 0 | | |
| Sucrose | 205 | | | 20 | | |
| Fructose | 22 | | | 0 | | |
| Cellulose, BW200 | 18.2 | | | 40.7 | | |
| Inulin | 6 | | | 13.5 | | |
| Menhaden Oil (200 ppm tBHQ) | 1 | | | 13.2 | | |
| Butter, Anhydrous | 54.1 | | | 7.4 | | |
| Flaxseed Oil | 1 | | | 6.1 | | |
| t-BHQ | 0.0047 | | | 0.0023 | | |
| Mineral Mix S10026 | 10 | | | 10 | | |
| Dicalcium Phosphate | 13 | | | 13 | | |
| Calcium Carbonate | 5.5 | | | 5.5 | | |
| Potassium Citrate, 1 H2O | 16.5 | | | 16.5 | | |
| Vitamin Mix V10001 | 10 | | | 10 | | |
| Biotin (1%) | 0.1 | | | 0.014 | | |
| Choline Bitartrate | 2 | | | 2 | | |
| Cholesterol | 1.5 | | | 0 | | |
| 50% Trans Resveratrol | 0 | | | 0.045 | | |
| Cellulose, BW200 (Insoluble) (g/kg) | 21 | | | 56.8 | | |
| Inulin (Soluble) (g/kg) | 6.9 | | | 18.9 | | |
| Total Fiber (g/kg) (includes starch) | 26.3 | | | 71 | | |
| Cholesterol (mg/kg) | 1935.9 | | | 213.8 | | |
| 50% Trans Resveratrol (mg/kg) | 0 | | | 62.8 | | |
| tBHQ (mg/3641 kcals) | 4.9 | | | 4.9 | | |
| Biotin (mg/3641 kcals) | 1.2 | | | 0.34 | | |
