# Supplementary material for: A modified Mediterranean-style diet enhances brain function via specific gut-microbiome-brain mechanisms
Source: Gut Microbes. 2024 Mar 6;16(1):2323752. doi: 10.1080/19490976.2024.2323752 (PMC10936641; doi:10.1080/19490976.2024.2323752)
Supplement: Supplemental Material [file KGMI_A_2323752_SM1749.zip › Supplementary Figures S1_10_KGMI_20231006_R1.docx]

#
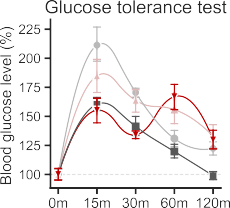

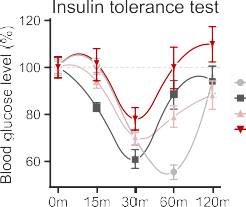
d


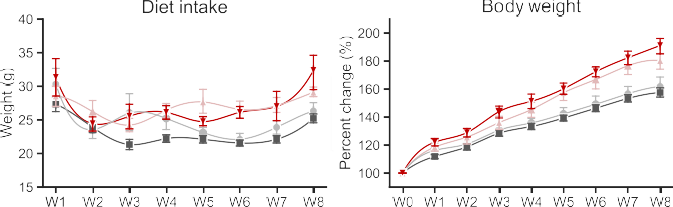


**a**

**b**

**c**

**#**

**#**

*****

**#**

*****

WT-WD WT-MkD AD-WD AD-MkD

#
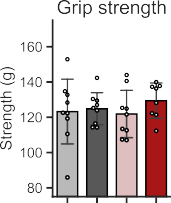
g


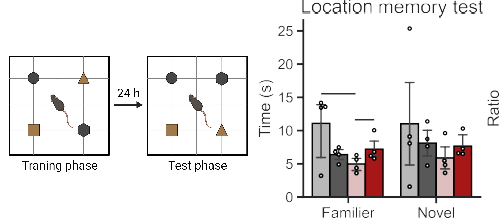

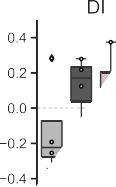


**f**

**#**

**#**


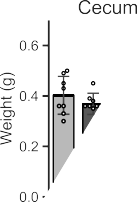


**e**


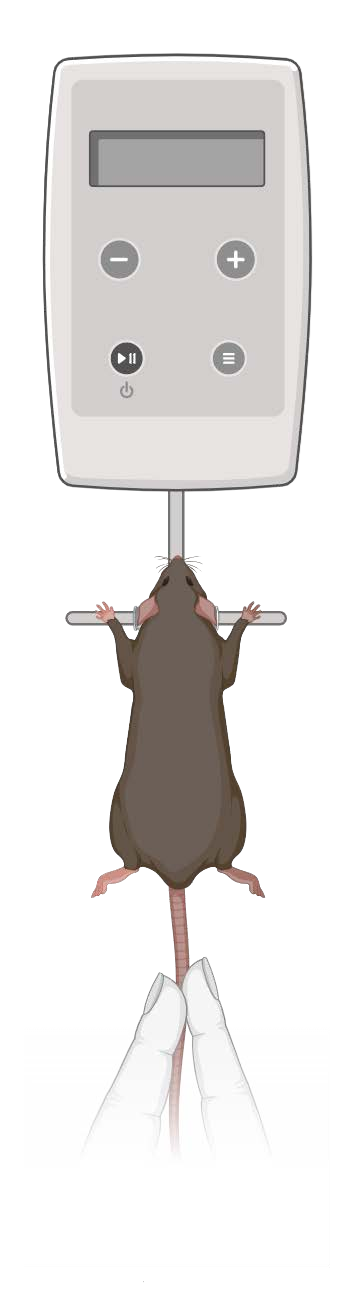


**Figure S1. Additional profiles of the experiment and measurements.** Overall trend depicting weekly (A) diet intake and (B) percent change in bodyweight. Percent changes in Blood glucose level over time (two hours) after (C) oral glucose injection and (D) intraperitoneal insulin injection. (E) Comparison of cecum weight. (F) Time spent near familiar

WT-WD WT-MkD AD-WD

AD-MkD

WT-WD WT-MkD AD-WD

AD-MkD

WT-WD WT-MkD AD-WD

AD-MkD

and novel locations, along with the discrimination index value in the location memory test (n=4/group). (G) Grip strength. The data presented in the graphs are the mean ± SD. Significance was determined by the one-way ANOVA test with post-hoc Dunn’s test. n=8-9 per group (except for location memory test). #<0.1; *<0.05. For the line plots, the

significance between groups was differentiated by color. Light gray color represents the difference between WT-WED and AD-WED and red; WT-MED and AD-MED.

# a

**e** Taxa

WT-WD WT-MkD

p-val

**f** Correlation coefficient (ρ)


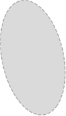

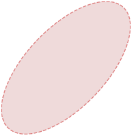

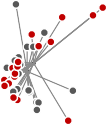

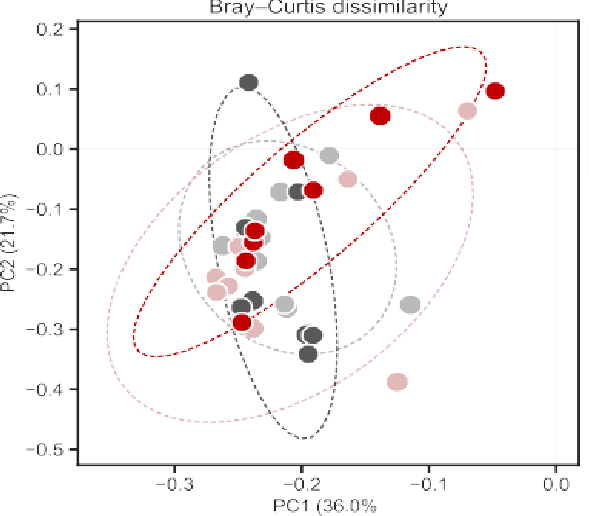

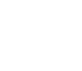

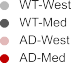


**b**


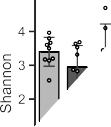

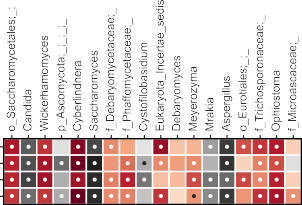

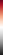


WT 0.469


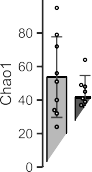
AD


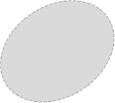

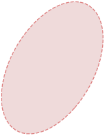

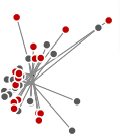


WT-WD WT-MkD AD-WD AD-MkD

WT-WD WT-MkD

WT-WD

WT-MkD

AD-WD AD-MkD

0.277

AD-WD AD-MkD

0.109 WD

MkD

0.784

**c** Phylum level **d**


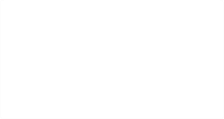

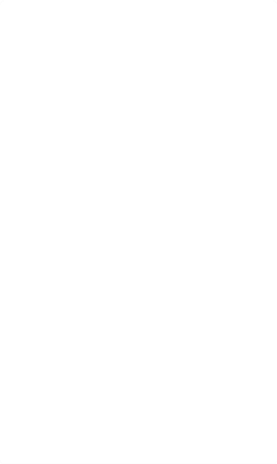

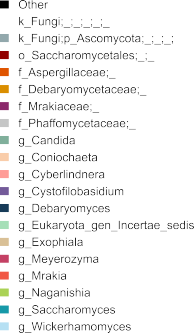


Genus level


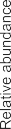


**Figure S2. Fungal community profile after 8-week intervention.** (A) Alpha-diversity was determined using the Shannon index and Chao1 index. Significance between groups was calculated using the non-parametric Kruskal-Wallis test. (B) PCoA analysis based on Bray-Curtis dissimilarity was used to represent the beta-diversity of each group, genotype, and diet. Significance was calculated using PERMANOVA with 999 random permutations. (C) Relative abundance of fungal composition at the (C) phylum and the (D) genus level. (E) Fungal composition differences between groups were analyzed using the Linear discrimination analysis (LDA) effect size (LEfSe) algorithm (LDA>3.0,

WT-WD

WT-MkD

AD-WD

AD-MkD

WT-WD

WT-MkD

AD-WD

AD-MkD

p-value<0.05). (F) The heatmap represents the taxa changes over time for each group. Spearman’s correlation was used, and significantly correlated taxa (p-value <0.05) are indicated with a ‘●’ sign. n=8-9 per group. The data presented in the graphs are the mean ± SD.

# a b


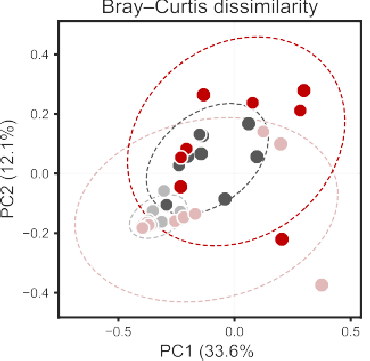


WT-WD WT-MkD

AD-WD AD-MkD

WT-WD

WT-MkD

AD-WD

AD-MkD

WT-WD

WT-MkD

AD-WD

AD-MkD

0.001

0.020

1.
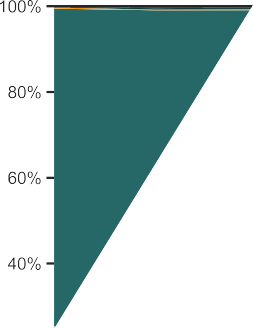
WT-WD WT-MkD AD-WD AD-MkD


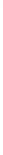

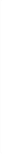

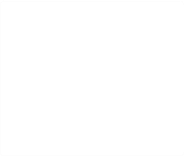

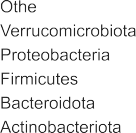


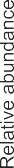


1.
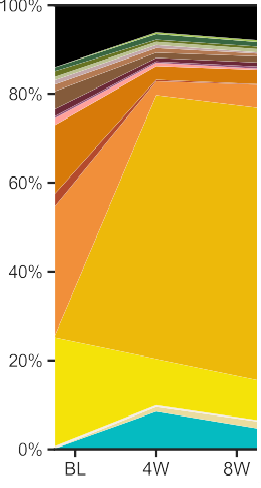
WT-WD WT-MkD AD-WD AD-MkD


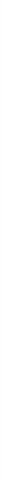

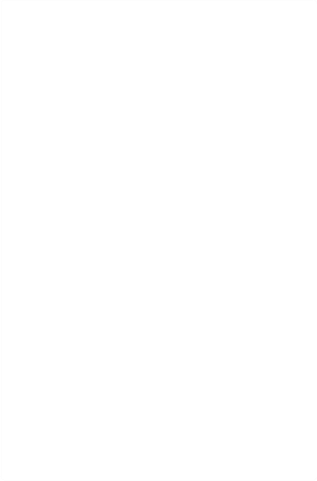

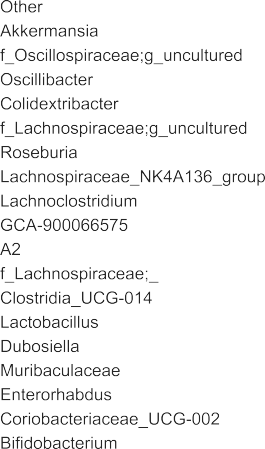


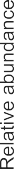


**Figure S3. Bacterial community profile after a 4-week intervention, and microbial compositional changes over time.** (A) Alpha-diversity was determined using the Shannon index and Chao1 index. Significance between groups was calculated using the non-parametric Kruskal-Wallis test. (B) PCoA analysis based on Bray-Curtis dissimilarity was used to represent the beta-diversity of each group, genotype, and diet. Significance was calculated using PERMANOVA with 999 random permutations. Area plots showing the changes in microbial composition over time at the (C) phylum and

the (D) genus level. n=8-9 per group. The data presented in the graphs are the mean ± SD.


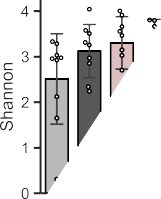
**a ****

**b**


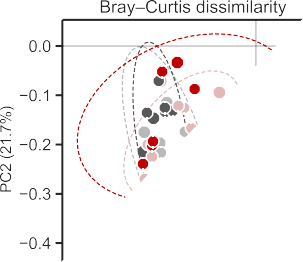
WT-WD WT-MkD

AD-WD AD-MkD

WT-WD

WT-MkD

AD-WD

AD-MkD

WT-WD

WT-MkD

AD-WD

AD-MkD

0.581

0.657

1. WT-WD WT-MkD AD-WD AD-MkD


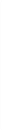

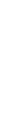

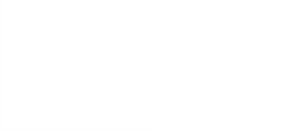

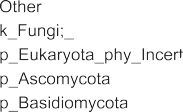


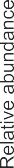


1.
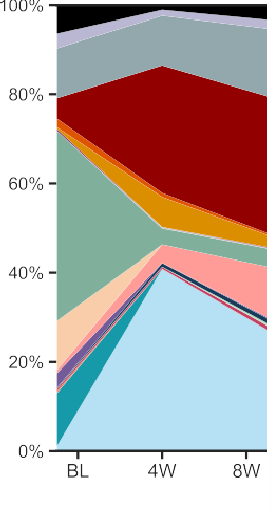
WT-WD WT-MkD AD-WD AD-MkD


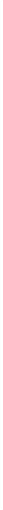

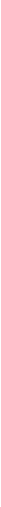

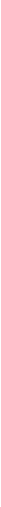

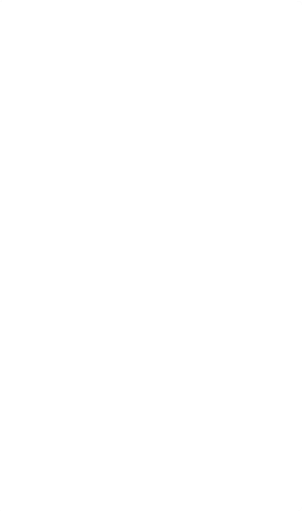

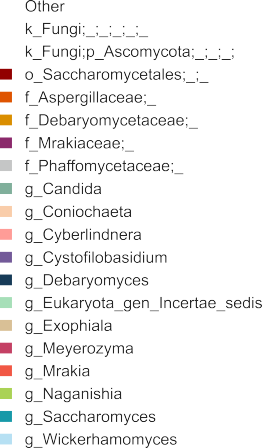


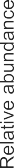


**Figure S4. Fungal community profile after a 4-week intervention, and microbial compositional changes over time.** (A) Alpha-diversity was determined using the Shannon index and Chao1 index. Significance between groups was calculated using the non-parametric Kruskal-Wallis test. (B) PCoA analysis based on Bray-Curtis dissimilarity was used to represent the beta-diversity of each group, genotype, and diet. Significance was calculated using PERMANOVA with 999 random permutations. Area plots showing the changes in microbial composition over time at the (C) phylum and

the (D) genus level. n=8-9 per group. The data presented in the graphs are the mean ± SD.

KEGG Pathway

WT-WD WT-MkD wi.eBH

KEGG Pathway

AD-WD AD-MkD wi.eBH


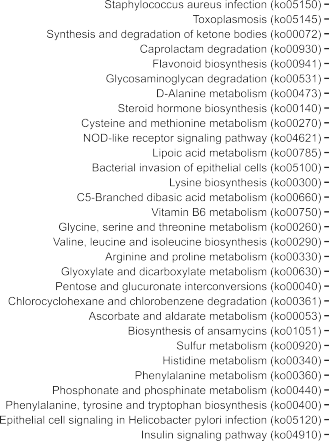

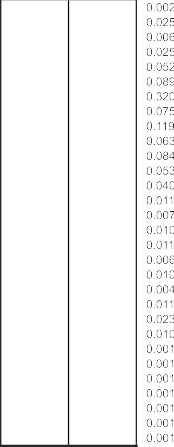

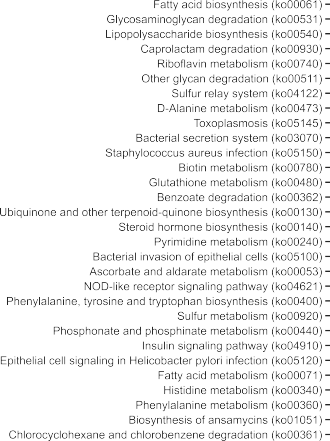

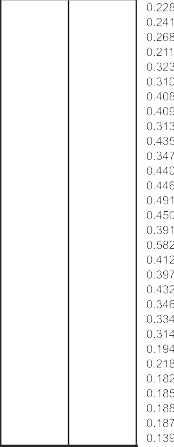


**Figure S5. Differences in predicted KEGG pathway between diets.** ALDEx2 was used to measure the differences between diets. Significance was determined using Welch’s t-test, and p-values were adjusted for false discovery rate (FDR) using the Benjamini-Hochberg adjustment.

**a WT**


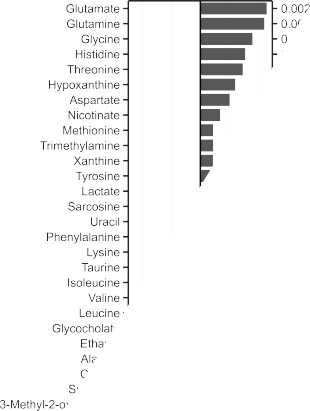
WD MkD

p-val

**AD**

WD MkD


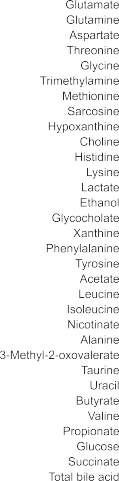

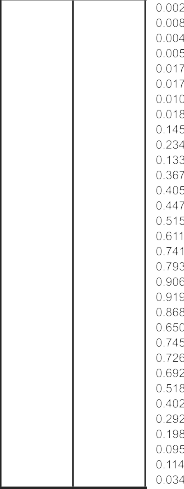


**b**

p-val


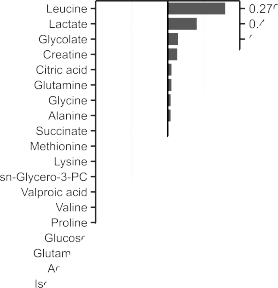


**WT**

WD MkD

p-val

**AD**

WD MkD


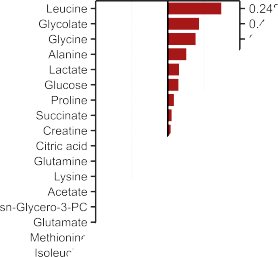


p-val

**Figure S6. Differences in metabolite abundance between diets.** ALDEx2 was used to measure the differences between diets. Significance was determined using Welch’s t-test, and p-values were adjusted FDR using the Benjamini-Hochberg adjustment.

**a**

**WT-WD WT-MkD AD-WD AD-MkD**

kDa


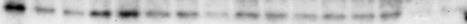

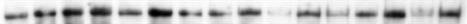


23

225

31


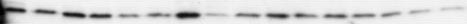

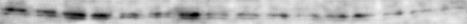

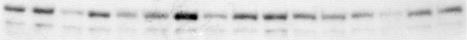


24

55

42


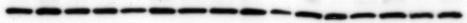


**b**


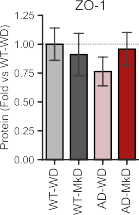

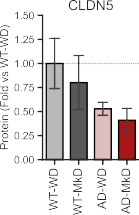
CLDN5 ZO-1

IL-1β

IL-6 TNF-α

β-actin


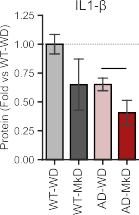


**#**


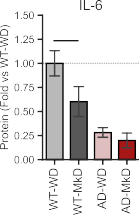


**#**


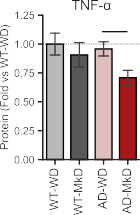


*****

**Figure S7. Protein quantification for key tight junction proteins and inflammatory markers in the brain.** (a) The protein levels of tight junction proteins (CODN5 and ZO-1) and inflammatory markers (IL-1β, IL-6, and TNF-α) in the brain were quantified by Western blot. (b) The protein levels presented by fold change calculated compared to WT-WD group. Significance is determined using unpaired t-tests between WT-WD and WT-MkD, as well as between AD-WD and AD-MkD. n=4 per group. #p<0.1; *p<0.05. WT: wild-type; AD: Alzheimer’s disease (APP/PS1 transgenic) mice; MkD: Mediterranean-ketogenic diet; WD: Western-style diet.


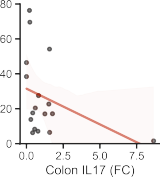
**a** ρ=-0.534 p=0.027 ρ=-0.547 p=0.028 ρ=-0.460 p=0.063 **b**


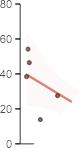

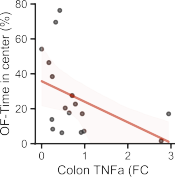

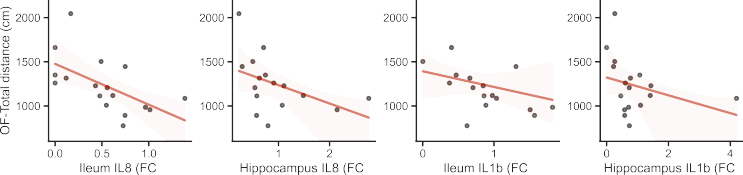


ρ=-0.658 p=0.004

ρ=-0.532 p=0.034

ρ=-0.453 p=0.068 ρ=-0.400 p=0.112

ρ=-0.432 p=0.094


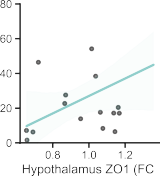


ρ=0.407 p=0.105


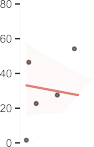

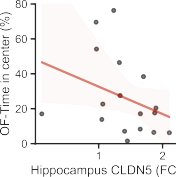


ρ=-0.444 p=0.074

ρ=-0.406 p=0.119


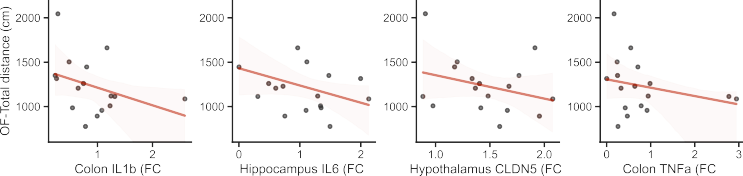


ρ=-0.365 p=0.149

ρ=-0.365 p=0.165

ρ=-0.350 p=0.168

**c** ρ=0.404 p=0.033 ρ=-0.342 p=0.069


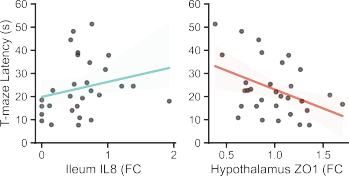


**d** ρ=-0.411 p=0.027 ρ=-0.411 p=0.030


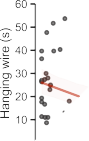

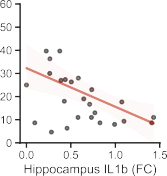


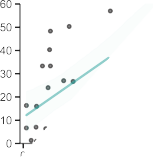

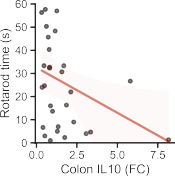
**e** ρ=-0.377 p=0.048 ρ=0.343 p=0.074


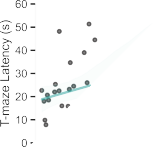


ρ=0.329 p=0.094


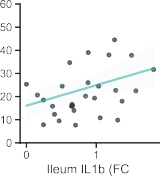


ρ=0.304 p=0.109


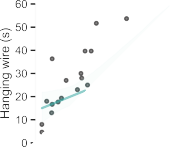


ρ=0.343 p=0.074


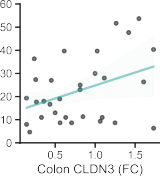


ρ=0.254 p=0.175


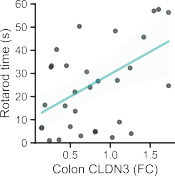


ρ=0.311 p=0.095


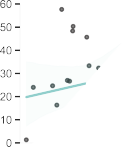


ρ=0.269 p=0.159

**Figure S8. Spearman’s correlational analysis results between gene expression and behavior assay results.** Correlation between tight-junction proteins and inflammatory markers in the gut and brain and (a) time spent in the center, (b) total traversed distance in the open-field test, (c) latency time of the T-maze test, (d) time in the hanging-wire test, and (e) time in the rotarod test.

# b


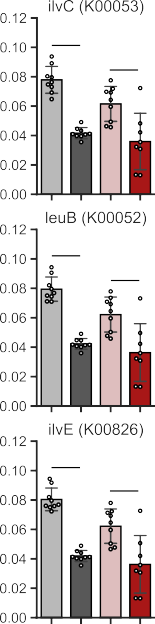


*******

******

*******

******

*******

******


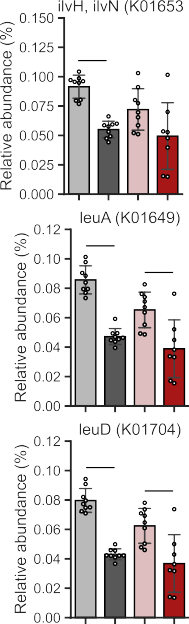

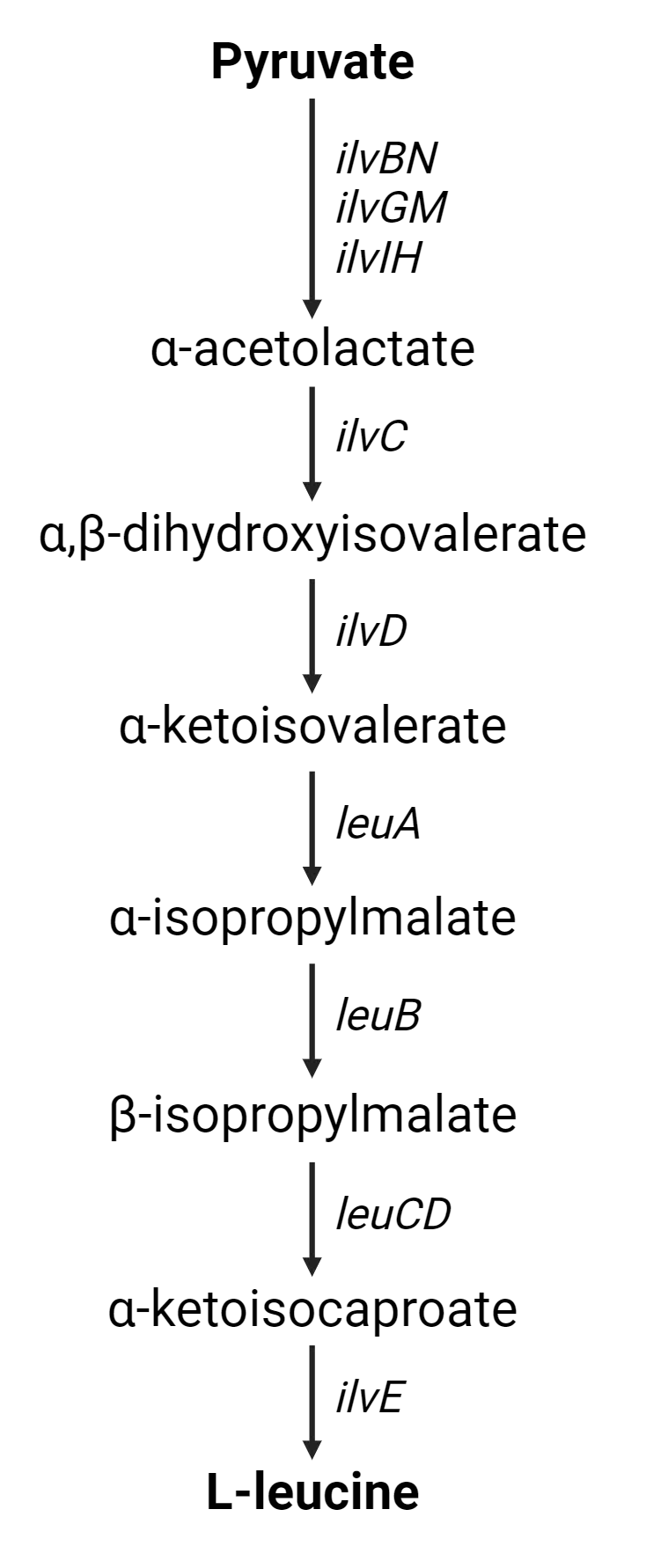


**a**

*******

*******

******

*******

******


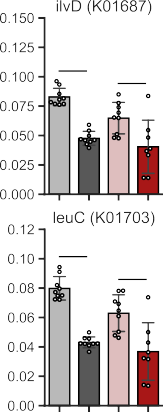


*******

*****

*******

******


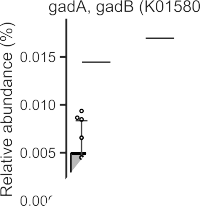


******

******

WT-WD WT-MkD AD-WD

AD-MkD

WT-WD WT-MkD AD-WD

AD-MkD

**Figure S9. The microbial biosynthetic pathway of lactate and leucine, and predicted abundance of genes**

WT-WD

WT-MkD AD-WD

AD-MkD

WT-WD WT-MkD AD-WD

AD-MkD

**associated with leucine biosynthesis.** (A) Bacterial biosynthesis pathway and associated genes for leucine and the predicted relative abundance of genes (KO orthology). (B) The predicted relative abundance of glutamate

decarboxylase (K01580) gene. The data presented in the graphs are the mean ± SD. Significance was determined

using unpaired t-tests between WT-WED and WT-MKD, as well as between AD-WED and AD-MKD. * p<0.05; ** p<0.01,

*** p<0.001.

**a** ρ=-0.547 p=0.002 ρ=-0.415 p=0.028 ρ=-0.301 p=0.120 ρ=0.294 p=0.129


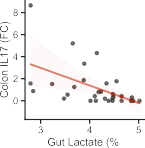

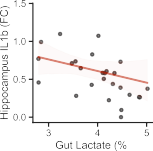

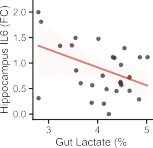

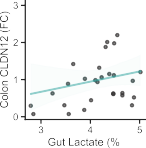


**c** ρ=0.502 p=0.007 ρ=-0.405 p=0.029 ρ=-0.374 p=0.050 ρ=0.374 p=0.055

ρ=-0.279 p=0.151

ρ=0.362 p=0.059 ρ=-0.315 p=0.096

**b** ρ=-0.456 p=0.066 ρ=0.444 p=0.076 ρ=-0.394 p=0.118 ρ=-0.379 p=0.147

**d** ρ=0.647 p=0.005 ρ=-0.631 p=0.009 ρ=-0.522 p=0.032 ρ=-0.500 p=0.041

ρ=-0.424 p=0.090 ρ=0.402 p=0.110 ρ=0.373 p=0.141 ρ=0.370 p=0.144

**e** ρ=-0.423 p=0.020 ρ=0.502 p=0.040 ρ=-0.301 p=0.106 ρ=0.378 p=0.039 ρ=0.353 p=0.164

**Figure S10. Spearman’s correlational analysis results between gut and serum lactate/leucine level and gene expression and behavior assay results.** Correlation between tight-junction proteins and inflammatory markers in the gut and brain and (a) gut lactate, (b) serum lactate, (c) gut leucine, and (d) serum leucine. (e) Spearman correlational analysis results between the outcomes of behavior assays and the level of gut lactate, gut leucine, and serum leucine.
